# Regulatory Mechanisms of L-Lactic Acid and Taste Substances in Chinese Acid Rice Soup (Rice-Acid) Fermented With a *Lacticaseibacillus paracasei* and *Kluyveromyces marxianus*

**DOI:** 10.3389/fmicb.2021.594631

**Published:** 2021-05-21

**Authors:** Na Liu, Likang Qin, Song Miao

**Affiliations:** ^1^Key Laboratory of Plant Resource Conservation and Germplasm Innovation in Mountainous Region (Ministry of Education), Collaborative Innovation Center for Mountain Ecology & Agro-Bioengineering (CICMEAB), College of Life Sciences/Institute of Agro-Bioengineering, Guizhou University, Guiyang, China; ^2^Teagasc Food Research Centre, Moorepark, Fermoy, Ireland; ^3^School of Liquor and Food Engineering, Guizhou University, Guiyang, China

**Keywords:** rice-acid, L-lactic acid, organic acid, taste substances, transcriptomics, regulatory mechanism

## Abstract

Rice-acid has abundant taste substances and health protection function due to the various bioactive compounds it contains, including organic acids. L-lactic acid is the most abundant organic acid in rice-acid, but the regulatory mechanisms of L-lactic acid accumulation in rice-acid are obscure. In this study, we analyzed the dynamic changes in organic acids and taste substances in rice-acid in various fermentation phases and different inoculation methods. We identified the key genes involved in taste substance biosynthesis by RNA-Seq analysis and compared the data of four experimental groups. We found that the interaction of the differences in key functional genes (L-lactate dehydrogenase and D-lactate dehydrogenase) and key metabolism pathways (glycolysis, pyruvate metabolism, TCA cycle, amino acid biosynthesis, and metabolism) might interpret the accumulation of L-lactic acid, other organic acids, and taste substances in rice-acid fermented with *Lacticaseibacillus paracasei*. The experimental data provided the basis for exploring regulatory mechanisms of taste substance accumulation in rice-acid.

## Introduction

As a traditional sour food of the Miao and Dong Nationalities, sour soup is popular in China because of its unique taste and flavor. Sour soup mainly includes “acid rice soup” (rice-acid) prepared with rice and water and “acid tomato soup” prepared with tomato or red pepper. Rice-based fermented cereal products are widely loved by consumers in the world, such as fermented rice vinegar, rice wine, and rice noodle. Rice-acid, as another rice-based fermented food, has unique taste substances and various physiologically active substances. Furthermore, it is believed that rice-acid has anti-fatigue, anti-aging, and immunity regulation properties alongside probiotic microbiota and can be used to adjust the human intestinal micro-ecological balance and prevent digestive diseases (Yuan, 2010).

The flavor quality of fermented foods can be improved by optimizing the microbiota structure and regulating the substance metabolism (Zang et al., 2020). Lactic acid is the main flavor component and the key nutritional component in lactic acid foods fermented with various lactic acid bacteria (LAB). However, the human body has only L-lactic acid dehydrogenase, which can only catalyze L-lactic acid. The excessive intake of D-lactic acid or DL-lactic acid may cause metabolic disorders and adverse reactions such as acidosis in the human body (Singh et al., 2019). It is, therefore, necessary to explore the metabolism pathway of L-lactic acid. Yeasts are widely used to ferment foods due to their contributions to the flavors of beverage and fermented cereal foods. LAB and yeasts in fermented foods interact with each other mostly in the metabolism of carbohydrates and nitrogen via stimulatory or inhibitory compounds (Minervini et al., 2014). In recent years, the composite fermentation systems of LAB and yeasts have been used to adjust the characteristics of acid, taste, and aroma. Du et al. (2018) explored the formation of ethyl carbamate in Chinese Moutai-flavor Liquor inoculated with LAB and nonconventional yeasts. Romanens et al. (2019) screened and selected the LAB and yeast strains in cocoa bean fermentation with a suitable flavor and quality. Both LAB and yeasts affect each other through the production of some metabolic products. The regulation mechanisms of organic acid metabolism or taste substance metabolism in these LAB-yeast cooperative fermentation systems, however, have not been reported. The mechanisms underlying L-lactic acid biosynthesis in rice-acid fermentation remain unclear.

The combination of RNA sequencing technologies, metabolic modeling, and physiological indexes offers a powerful tool to study the global response of cells to environmental changes and identify the regulatory mechanisms of targeted metabolites (Wang et al., 2019; Yuan et al., 2019). Actively expressed genes can be explored through transcriptome sequencing of the cDNA after RNA reverse transcription. The transcriptome sequencing can reveal actively expressed genes within a specific period and space, which can be associated with ongoing metabolomic changes as well as the formation of flavors and tastes in fermented foods (Chen et al., 2017). Importantly, the global response of LAB under yeast culture conditions should be studied and RNA sequencing can be used to obtain a global vision of the up- and down-regulated genes (Sun et al., 2019). Aiming at the key flavors (organic acids and taste substances), based on our previous metabolite data, we analyzed the biosynthesis pathways of organic acids and taste substances in the rice-acid fermentation process through the joint application of transcriptomics and metabolite data analysis.

In the study, we fermented rice-acid with *Lacticaseibacillus paracasei* (previously *L. paracasei*, Zheng et al., 2020) and *Kluyveromyces marxianus* (*K. marxianus*) in order to gain suitable organic acids and taste substances. Interestingly, *L. paracasei* has a good effect on the flavor and is usually found in the human intestinal tract or other natural habitats such as cereal products and plant materials. *L. paracasei* with some probiotic properties, including H4-11, has been used in the production of functional foods to improve flavors and extend the shelf life (Mantzourani et al., 2019). It was demonstrated that in fermentation foods, *K. marxianus* could metabolize rice into water, sucrose, glucose, fructose, gum, proteins, minerals, vitamins, beneficial compounds, etc. (Ortiz-Basurto et al., 2008). We attempted to investigate the biological regulation of *L. paracasei* during the maturation of rice-acid by using transcriptomics. Four pairwise comparisons were set in the experiment, including different fermentation time (1 and 3 days) and different inoculation methods (single inoculation of *L. paracasei* and mixed inoculation of *L. paracasei* and *K. marxianus*), in order to investigate the crucial genes of *L. paracasei* for the production of organic acids and taste substances in different rice-acid fermentation processes. The study aims to investigate the molecular mechanisms of formation and metabolic pathways of flavors in rice-acid fermented with *L. paracasei* under the conditions of different times and inoculation methods.

## Materials and Methods

### Preparation Methods of Fermented Rice-Acid

Two strains of *L. paracasei* H4-11 (*L. paracasei* H4-11, CCTCC 2021074) and *K. marxianus* L1-1 (*K. marxianus* L1-1, CCTCC 2021073) were isolated from the traditional fermented rice-acid with high contents of acid and aroma compounds and used in the fermentation experiments (Liu et al., 2020a, b). First, *L. paracasei* H4-11 was cultivated at 37°C for 48 h in MRS medium. *K. marxianus* L1-1 was cultivated at 30°C for 72 h in PDA medium. Single colonies of *L. paracasei* H4-11 and *K. marxianus* L1-1 were selected. Selected colonies of *L. paracasei* H4-11 were cultivated into MRS broth medium at 37°C for 16 h. Selected colonies of *K. marxianus* L1-1 were cultivated into PDB broth medium at 30°C for 24 h. Then, the two broth media were centrifuged at 3000 rpm for 5 min. Cells were washed three times with saline water containing 0.85% (*w/v*) NaCl and finally resuspended in saline water. The resuspended cells were used to ferment rice-acid. The cell densities of *L. paracasei* H4-11 and *K. marxianus* L1-1 were 8.99 log CFU/mL and 7.69 log CFU/mL, respectively.

Rice-acid was fermented according to the following method. Firstly, the selenium rice (Danzhai, Guizhou, China) was broken with a high-speed pulverizer and sieved twice with an 80-mesh sieve to prepare rice flour. Then, water was added into rice flour according to the proportions of 8.0% rice flour and 92.0% water and boiled under stirring conditions to obtain rice soup. Then, boiled rice soup was gelatinized for 30 min in a water bath at 60°C under stirring conditions to prevent uneven gelatinization and local deterioration of the gelatinization solution. Then 1.0% α-amylase was added into gelatinized rice soup for 30-min liquefaction at 90 °C. Then, 0.02% saccharifying enzyme (glucoamylase) was added for 2-h saccharification at 60°C. The mixture was sterilized at 90°C for 20 min. After the rice soup was cooled to about 30°C, *K. marxianus* L1-1 and *L. paracasei* H4-11 were added according to the cell densities of 5.69 log CFU/mL and 7.89 log CFU/mL. Then, the prepared raw materials were poured into the sterilized rice-acid fermenter and sealed immediately. Finally, the mixture was fermented in a constant-temperature incubator at 30°C for 3 day. We compared different fermentation stages (1 and 3 days) in two rice-acid samples inoculated with *L. paracasei* H4-11, namely, L1 and L3 days. The other two rice-acid samples were inoculated with the mixed culture (*L. paracasei* H4-11 and *K. marxianus* L1-1) for 1 and 3 days, namely, LY 1 and LY 3 days.

### Determination of the Cell Densities of *L. paracasei* H4-11 and *K. marxianus* L1-1

Four samples (L1, L3, LY 1, and LY 3 days, 5 g) were mixed separately with 45 mL of saline water containing 0.85% (*w/v*) NaCl and shaken at 150 rpm for 30 min at room temperature. Then, the mixed rice-acid was serially diluted by 10^5^ times with saline water containing 0.85% (*w/v*) NaCl and spread on MRS agar medium and PDA medium, respectively. *L. paracasei* H4-11 was cultured in MRS medium at 37°C for 48 h. *K. marxianus* L1-1 was cultured in PDA medium at 30°C for 72 h. The cell densities of *L. paracasei* H4-11 and *K. marxianus* L1-1 were determined in 1 and 3 days, respectively.

### Determination of Organic Acids and Taste Substances

After settlement, the samples of rice-acid were filtered with double-layer filter paper. The obtained filtrate was filtered through a 0.22-μm microporous membrane and then passed through a ZORBAX SB-AQ solid-phase cartridge for HPLC analysis (equipped with G1329B autosampler, G1311C quaternary low-pressure ladder, G1316A column oven, and G1315D diode array UV–visible light detector) with ZORBAX SB-Aq column (4.6 × 250 mm, 5 μm, American Agilent Corporation) according to the following parameters: 0.02 mol/L NaH_2_PO_4_ solution as the mobile phase (pH 2.7), the injection volume (10 μL), flow rate (0.9 mL/min), column temperature (35°C), and detector wavenumber (UV 210 nm). The purity of L-lactic acid was determined according to the method of Moon et al. (2012). L-lactic acid concentration was measured with Amplite^TM^ Colorimetric l-Lactate Assay Kit (AAT Bioquest Inc., United States). Firstly, 0.5, 1.0, 2.0, 5.0, and 10.0 mL of organic acid standards (oxalic acid, tartaric acid, malic acid, acetic acid, citric acid, and succinic) were taken, respectively, diluted to a volume of 25 mL with ultrapure water in a volumetric flask, and filtered through a 0.22-μm aqueous phase filter membrane in order to obtain organic acids with different concentrations. With acid standard solutions, a standard curve with peak area versus concentration was plotted to obtain the linear range regression equation and correlation coefficient and calculate the concentration of each organic acid in rice-acid. In this way, the concentrations of oxalic acid, tartaric acid, malic acid, acetic acid, citric acid, and succinic acid in rice-acid samples were determined.

Rice-acid filtrate (8 mL) was diluted to a volume of 80 mL and analyzed by an electronic tongue (Insent SA-402B, Atsugi-chi, Japan) to determine taste substances (sourness, bitterness, astringency, umami, richness, saltiness, aftertaste-A, and aftertaste-B) according to the method of Qiu et al. (2015). The electronic sensor was cleaned in a cleaning solution for 90 s. The first reference solution was added to clean the sensor for 120 s. The second reference solution was added to clean the sensor for 120 s. After cleaning, the sensor was zeroed to the equilibrium position for 30 s and then placed in the sample cup for 30 s. After the test, the sensor was sequentially washed in two reference solutions for 3 s. Each sample was tested four times. After excluding the first repeated result, the average of the remaining three results was used in the subsequent analysis.

### Transcriptome Sequencing and Data Analysis

#### RNA Extraction and Library Construction for Transcriptome Analysis

The four samples (L1, L3, LY1, and LY3 days, and each sample had three biological repeats) were filtered to get the cells and then centrifuged at 10,000 g for 10 min at 4 °C to obtain the *L. paracasei* cells, which was put in liquid nitrogen quickly and then stored in a refrigerator at −80°C. The RNA extraction of *L. paracasei* H4-11 cells, library construction, RNA-seq, and primary data analysis was performed by PTM BIO Co., Ltd. (Hangzhou, China). The bacterial powder was suspended in 1 mL of Trizol reagent (Invitrogen, Carlsbad, CA, United States) and manipulated according to the manufacturer’s instructions. The RNA samples were resuspended in RNase-free water (Thermo Fisher Scientific, Waltham, MA, United States). RNA degradation and contamination were monitored on 1% agarose gels. Then, RNA purity was checked with NanoPhotometer^®^ spectrophotometer (IMPLEN, CA, United States). RNA concentration was measured with Qubit^®^ RNA Assay Kit in Qubit^®^ 2.0 Flurometer (Life Technologies, CA, United States). RNA integrity was assessed by using the RNA Nano 6,000 Assay Kit of the Agilent Bioanalyzer 2,100 system (Agilent Technologies, CA, United States).

A total of 3 μg RNA per sample was used as the input material for the RNA sample preparation. Firstly, ribosomal RNA was removed by Epicentre Ribo zeroTM rRNA Removal Kit (Epicentre, United States) and rRNA-free residue was cleaned by ethanol precipitation. Subsequently, sequencing libraries were generated with the rRNA-depleted RNA by NEBNext^®^ UltraTM Directional RNA Library Prep Kit for Illumina^®^ (NEB, United States) according to the manufacturer’s recommendations. Briefly, fragmentation was carried out with divalent cations at an elevated temperature in NEBNext First Strand Synthesis Reaction Buffer (5X). First-strand cDNA was synthesized with random hexamer primers and M-MuLV Reverse Transcriptase (RNase H-). Second strand cDNA synthesis was subsequently performed with DNA Polymerase I and RNase H. In the reaction buffer, the dTTP of dNTPs were replaced by dUTP. The remaining overhangs were converted into blunt ends via exonuclease/polymerase activities. After adenylation of 3′ ends of DNA fragments, the NEBNext Adaptor with a hairpin loop structure was ligated to prepare for hybridization. In order to preferentially select cDNA fragments of 250∼300 bp in length, the library fragments were purified with the AMPure XP system (Beckman Coulter, Beverly, United States). Then 3 μL of USER Enzyme (NEB, United States) was incubated with size-selected and adaptor-ligated cDNA at 37°C for 15 min, followed by 5-min incubation at 95°C before PCR. PCR was then performed using Phusion High-Fidelity DNA polymerase, Universal PCR primers, and the Index (X) Primer. At last, PCR products were purified (AMPure XP system) and the library quality was assessed on the Agilent Bioanalyzer 2,100 system.

The clustering analysis of the index-coded samples was performed on cBot Cluster Generation System with Novaseq 6000 PE Cluster Kit (Illumia) according to the manufacturer’s instructions. After the generation of clusters, the library preparations were sequenced on an Illumina Novaseq 6000 platform and 150-bp paired-end reads were generated.

#### De novo Transcriptome Assembly and Annotation

Raw reads were generated by a sequencing machine and transformed by base calling. Clean data (clean reads) were obtained by removing reads containing adapter, reads containing ploy-N, and low-quality reads from raw data. Q20, Q30, and GC content of clean reads were calculated. To obtain the clean reads, those reads were filtered with the procedure developed by PTM BIO Co. Ltd. (Hangzhou, China). Next, clean reads were de novo assembled into contig with the Trinity platform (v2.0.12). We selected TopHat as the mapping tool since TopHat could generate a database of splice junctions based on the gene model annotation file and a better mapping result than other non-splice mapping tools. The reference genome (*L. paracasei* ATCC 334) and gene model annotation files can be directly downloaded from the genome website^1^. Cuffquant and cuffnorm (v2.2.1) were used to calculate FPKMs (reads per kilobase of exon region per million mappable reads) of genes in each sample. The transcriptome sequencing was performed according to the method of Huang et al. (2019). The differentially expressed genes (DEGs) were identified in the four pairwise comparisons. DEGs were defined as the value of | Fold Change (FC) | >1.5 and a false discovery rate (FDR) < 0.01. Both Gene Ontology (GO) terms and Kyoto Encyclopedia of Genes and Genomes (KEGG) pathways with *q*-value ≤ 0.05 were significantly enriched in DEGs.

### Statistical Analysis

All experiments were conducted in triplicate. Data were represented as mean ± standard deviation. Duncan’s multiple range test and *t*-test were carried out to analyze significant differences in SPSS Version 20.0 (SPSS Inc., Chicago, IL, United States). *P* < 0.05 was considered to be statistically significant. In the analysis process of differential expression genes, the recognized Benjamini-Hochberg correction method was used to correct the significance *p*-value obtained from the original hypothesis test, and a FDR (FDR < 0.01) was used as the key indicator for screening differential expression genes. The correlations between organic acids (oxalic acid, tartaric acid, malic acid, acetic acid, citric acid, and succinic) and taste substances (sourness, bitterness, astringency, umami, richness, saltiness, aftertaste-A, and aftertaste-B) and the expressions of organic acid metabolism and transporter genes were measured with Pearson’s correlation coefficients (Jawad et al., 2020).

## Results

### Variations of the Cell Densities of *L. paracasei* H4-11 and *K. marxianus* L1-1 in the Fermentation Process of Rice-Acid

**FIGURE 1:**
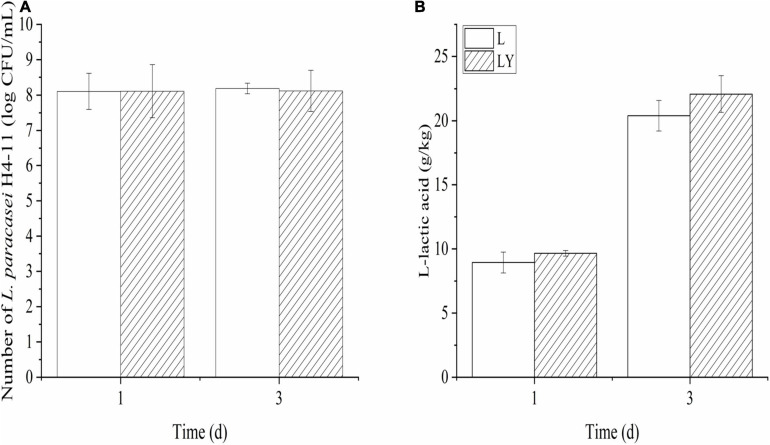
The variations of the number of *L. paracasei* **(A)** and the concentration of L-lactic acid **(B)** in the fermentation period of 1 and 3 days of rice-acid inoculated with *L. paracasei* and mixed-culture with *L. paracasei* and *K. marxianus* (L means rice-acid inoculated with *L. paracasei*, LY means rice-acid inoculated with *L. paracasei* and *K. marxianus*).

The cell densities of *L. paracasei* and *K. marxianus* in rice-acid inoculated with single starter and mixed starters increased in the fermentation period from 1 to 3 days (Figure 1A) and the increasing rate of *K. marxianus* L1-1 cells was faster than that of *L. paracasei* H4-11 cells. After 3-day fermentation, the cell density of *L. paracasei* in the group of single inoculation was higher (8.19 log CFU/mL) than that in the mixture of a single inoculation (8.12 log CFU/mL) (*P* > 0.05). Likely, the cell density of *K. marxianus* in the group of mixed inoculation (7.43 log CFU/mL) was lower than that in the group of single inoculation (7.61 log CFU/mL) (*P* > 0.05).

### Variations of Organic Acids in the Fermentation Process of Rice-Acid

**FIGURE 2:**
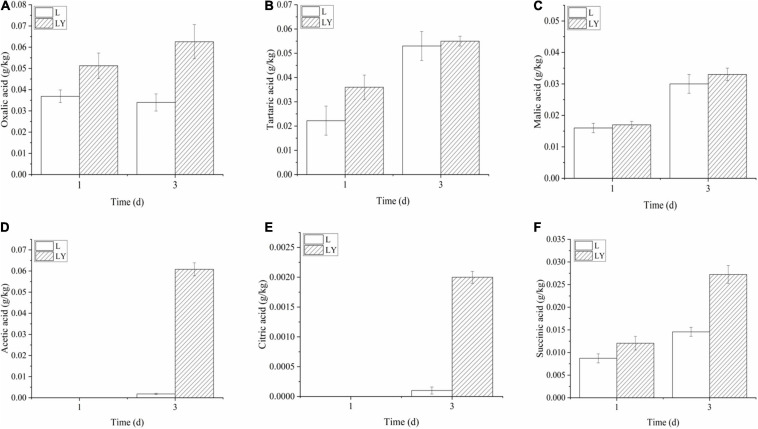
The variations of organic acids in the fermentation period of 1 and 3 days of rice-acid inoculated with *L. paracasei* and mixed-culture with *L. paracasei* and *K. marxianus* [L means rice-acid inoculated with *L. paracasei*, LY means rice-acid inoculated with *L. paracasei* and *K. marxianus*. Organic acids include oxalic acid **(A)**, tartaric acid **(B)**, malic acid **(C)**, acetic acid **(D)**, citric acid **(E)**, and succinic acid **(F)**].

The concentration of L-lactic acid in the group of mixed inoculation was 22.08 ± 2.42 g/kg after 3-dday fermentation (Figure 1B) and the optical purity of L-lactic acid was as high as 96%. The concentration of L-lactic acid in rice-acid inoculated with *K. marxianus* L1-1 was only 6.02 ± 1.67 g/kg. The other six organic acids showed different changing trends during the fermentation process (Figure 2). The values of malic acid and acetic acid increased in the fermentation process and the value of oxalic acid showed no significant variation. The values of three organic acids, including tartaric acid, citric acid, and succinic acid, slightly increased in the fermentation period from 1 to 3 days.

### Variations of Taste Intensity in the Fermentation Process of Rice-Acid

**FIGURE 3:**
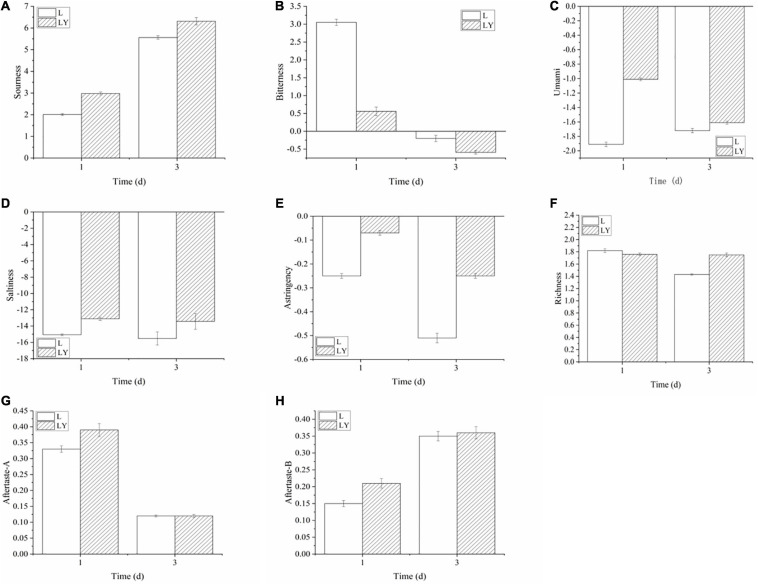
The variations of taste signal value of taste substances in the fermentation period of 1 and 3 days of rice-acid inoculated with *L. paracasei* and mixed-culture with *L. paracasei* and *K. marxianus* [L means rice-acid inoculated with *L. paracasei*, LY means rice-acid inoculated with *L. paracasei* and *K. marxianus*. Taste substances include sourness **(A)**, bitterness **(B)**, umami **(C)**, saltiness **(D)**, astringency **(E)**, richness **(F)**, aftertaste-A **(G)**, and aftertaste-B **(H)**].

The intensity variations of eight tastes measured by E-tongues within the three-day fermentation process decreased according to the following order: sourness > bitterness > umami > saltiness > astringency> richness > aftertaste-A > aftertaste-B (Figure 3). The group of mixed inoculation showed lower bitterness intensity than the group of single inoculation. The umami index showed a small difference between the group of single inoculation of *L. paracasei* H4-11 and the group of mixed inoculation. Interestingly, the mixed inoculation had smaller values of saltiness and astringency than single inoculation. Saltiness values of rice-acid showed slight differences between the groups of different inoculation methods due to the degradation of different proteins by different fermentation strains. The richness index in the group of mixed inoculation was higher than that in the group of single inoculation in the late stage. Aftertaste-A and aftertaste-B had a small influence on the flavor of rice-acid.

### RNA Sequencing Results and Differential Expressions of Genes

The four independent cDNA libraries (L1, L3, LY1, and LY3 days) constructed for high-throughput sequencing produced 7,824,981∼10,228,721 pair-end reads and 2,347,494,300∼3,068,616,300 clean reads after strict quality check and data filtering (Q20 bases > 97.54%, Q30 bases > 92.66%, G + C approximately 45.25%∼50.29%) (Supplementary Table 1, Supporting Information). The filtered clean sequence data were compared with the reference genome (*L. paracasei* ATCC 334). The percentage of the sequences that could be localized to the genome exceeded 90.00%, which suggested that the throughput and sequencing quality were high enough for further analysis. Moreover, we used Pearson′s correlation coefficient to analyze whether the differences depended on fermentation time and inoculation methods (mixed inoculation and single inoculation). R values in the three replicates of the same experiment were more than 0.989 (Supplementary Figure 1).

The comparison of different transcripts revealed that 1141 and 1094 genes were significantly differentially expressed in the period from L1 to L3 days and the period from LY1 to LY3 days, respectively. In addition, 601 significantly DEGs were found between the data of LY1 and L1 days, and 329 significantly DEGs were found between the data of LY3 and L3 days. The up-regulated and down-regulated genes accounted for approximately 50% (Supplementary Table 2).

### GO Enrichment Analysis

To further elaborate on the function of DEGs, we firstly conducted GO enrichment analysis. GO analysis of the DEGs showed the enrichment of major biological processes, molecular function, and cellular components (Supplementary Figure 2). The groups of different fermentation methods (single inoculation and mixed inoculation) were compared. The DEGs were enriched in catalytic activity and binding in terms of molecular functions. In terms of cellular components, most of the DEGs were enriched in four categories including membrane, membrane part, cell, and cell part.

Among the up-regulated and down-regulated DEGs, related biological processes mainly included the lysine biosynthetic process, oxidation-reduction process, NAD biosynthetic process, and purine nucleobase biosynthetic process. Related cellular components mainly included the ribosome, cytoplasm, and plasma membrane. Molecular functions mainly included structural components of ribosome and rRNA binding. The above terms were commonly enriched GO terms in the four pairwise comparisons (Supplementary Figure 3). Interestingly, there were more molecular function-related enriched GO terms in the fourth pairwise comparison (LY3 vs L3 days).

### Composite Analysis of KEGG Metabolism Pathways, Organic Acids, and Taste Substances

The top 20 significantly enriched pathways in the four pairwise comparisons were selected (Supplementary Figure 4). ABC transporters, ribosome, purine metabolism, amino sugar metabolism, and nucleotide sugar metabolism were enhanced in the four pairwise comparisons. These pathways were related to the general features of different transcriptomes of fermented rice-acid. The comparison results of different fermentation time (3 and 1 days) indicated that the enrichment of the differentially transcribed genes in the categories of beta-Lactam resistance, photosynthesis, nicotinate and nicotinamide metabolism, and oxidative phosphorylation were high in rice-acid only inoculated with *L. paracasei*, whereas the enrichment of the differentially transcribed genes in the categories of ribosomes, lysine biosynthesis, cysteine, and methionine metabolism, fatty acid biosynthesis and photosynthesis were high in rice-acid with mixed inoculation. The comparison results of different inoculation methods (single inoculation of *L. paracasei* and mixed inoculation) indicated that the enrichment of the differentially transcribed genes in the categories of alanine, aspartate and glutamate metabolism, aminoacyl-tRNA biosynthesis and TCA cycle were high in the first day, whereas the enriched differentially transcribed genes in the categories of ribosomes, histidine metabolism, purine metabolism, and ABC transporters were high in the third day.

**FIGURE 4:**
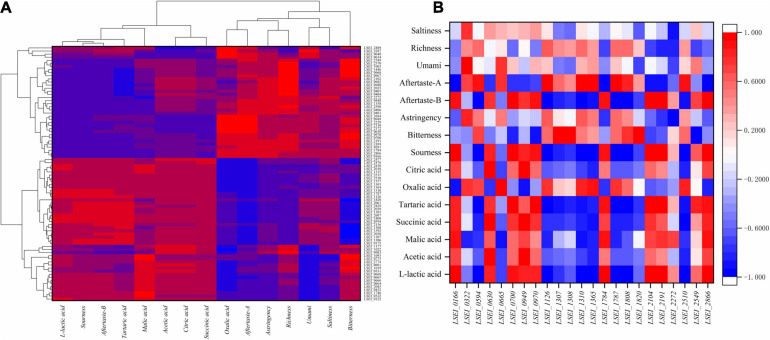
Correlations among the characteristic flavor (organic acids and taste substances) and gene expression **(A)**, Correlations among the characteristic flavor (organic acids and taste substances) and the key 23 genes **(B)**.

Moreover, we calculated Pearson correlation coefficients with the expression data obtained at different fermentation times (Figure 4). The expression levels of several genes (LSEI_0166, LSEI_1784, LSEI_2104, LSEI_2191, and LSEI_2866) had significant and positive correlation with L-lactic acid concentration (*r* = 0.983^∗^, 0.974^∗^, 0.966^∗^, 0.951^∗^, and 0.992^∗∗^) in rice-acid fermented with the single inoculation of *L. paracasei* and the mixed inoculation. The expression levels of several genes (LSEI_0594, LSEI_1126, LSEI_1310, LSEI_1365, LSEI_1787, LSEI_1808, LSEI_2410, and LSEI_2510) had the significant and negative correlation with L-lactic acid concentration (*r* = −0.981^∗^, −0.997^∗∗^, −0.998^∗∗^, −0.956^∗^, −0.999^∗∗^, −0.955^∗^, −0.961^∗^, and −0.960^∗^) in fermented rice-acid. The expression levels of the two genes of LSEI_0630 and LSEI_0949 had a significant and positive correlation with the concentrations of acetic acid, succinic acid, and citric acid. The expression levels of the gene LSEI_2477 had a significant and negative correlation with the concentrations of acetic acid, succinic acid, and citric acid. Similarly, the expression levels of the three genes (LSEI_0700, LSEI_2104, and LSEI_2866) had a significant and positive correlation with sourness intensity (*r* = 0.950^∗^, 0.984^∗^, and 0.961^∗^) in fermented rice-acid. The expression levels of the gene LSEI_2549 had a positive correlation with L-lactic acid concentration and sourness intensity and LSEI_2549 had a significant and negative correlation with bitterness intensity (*r* = 0.953^∗^). The expression levels of the two genes (LSEI_0401 and LSEI_2272) had a significant and negative correlation with astringency intensity (*r* = −0.964^∗^ and −0.974^∗^). The expression levels of the three genes (LSEI_0322, LSEI_0775, and LSEI_1746) had a significant and positive correlation with umami intensity (*r* = 0.958^∗^, 0.966^∗^, and 0.965^∗^) in fermented rice-acid.

#### Genes Involved in Ribosomes, ABC Transporters, and Purine Metabolism

The comparison results of different fermentation time (3 and 1 days, Supplementary Table 3) indicated that ribosomal transcription levels of the genes encoding large subunit ribosomal protein L32 (LSEI_1358) and large subunit ribosomal protein L7/L12 (LSEI_2272) were up-regulated in the group of the single inoculation with *L. paracasei*, whereas the genes encoding large subunit ribosomal protein L17 (LSEI_2476) and small subunit ribosomal protein S12 (LSEI_2510) were down-regulated in the group of the mixed inoculation. The comparison results of two inoculation methods (mixed inoculation and single inoculation of *L. paracasei*) indicated that the gene LSEI_2272 was down-regulated on the first day and the third day in the rice-acid fermentation process, whereas the genes LSEI_2476 and LSEI_2510 were down-regulated on the third day of rice-acid fermentation.

The comparison results of different fermentation time (3 and 1 days, Supplementary Table 3) indicated that ABC transporter transcription levels of the gene encoding L-cystine transport system substrate-binding protein (LSEI_0601) and the gene encoding D-methionine transport system substrate-binding protein (LSEI_1177) were up-regulated, whereas ABC transporter transcription levels of some genes encoding oligopeptide transport system substrate-binding protein (LSEI_0175), ATP-binding cassette, subfamily B, multidrug efflux pump (LSEI_1592), and oligopeptide transport system ATP-binding protein (LSEI_2061) were down-regulated in the group of the single inoculation of *L. paracasei*. The five genes except LSEI_1592 were down-regulated in the group of the mixed inoculation. The comparison results of different inoculation methods (mixed inoculation and single inoculation of *L. paracasei*) indicated that the changes of transcription levels of key genes in the first day of rice-acid fermentation were similar to those of the first pairwise comparison (L3 vs L1 days). The genes encoding L-cystine transport system substrate-binding protein (LSEI_0601) and D-methionine transport system substrate-binding protein (LSEI_1177) were up-regulated in the first and third pairwise comparisons.

The comparison results of different fermentation time (Supplementary Table 3) indicated that purine metabolism transcription levels of the genes encoding putative integral membrane protein with a TlyC-like hemolysin domain (LSEI_2229) was up-regulated, whereas the transcription level of the gene encoding adenylosuccinate synthase (LSEI_0122) was down-regulated in the group of the single inoculation of *L. paracasei*. The genes encoding phosphoglucomutase (LSEI_0949), ribonucleoside-triphosphate reductase (LSEI_2287), and the gene (LSEI_2229) were up-regulated and the genes encoding adenine phosphoribosyltransferase (LSEI_1557), phosphoribosylamine-glycine ligase (LSEI_1746), DNA-directed RNA polymerase subunit alpha (LSEI_2477) and DNA-directed RNA polymerase subunit beta′ (LSEI_2515) and the gene (LSEI_0122) were down-regulated in the group of the mixed inoculation. The comparison results of inoculation methods (single inoculation of *L. paracasei* and mixed inoculation) indicated that the genes LSEI_0122 and LSEI_1746 were up-regulated on the first day of rice-acid fermentation. On the third day of rice-acid fermentation, the genes of LSEI_0122, LSEI_0949, LSEI_1746, LSEI_2229, and LSEI_2287 were up-regulated and the genes of LSEI_1557, LSEI_2477, and LSEI_2515 were down-regulated.

#### Genes Involved in Amino Sugar and Nucleotide Sugar Metabolism and Starch and Sucrose Metabolism

The key DEGs involved in amino sugar and nucleotide sugar metabolism and starch and sucrose metabolism were related to energy production and conversion, carbohydrate transport and metabolism, cell wall/membrane/envelope biogenesis, and general function prediction. We further confirmed 21 DEGs at corresponding positions of amino sugar and nucleotide sugar metabolism (Supplementary Table 4). The comparison results of the different fermentation times confirmed 10 up-regulated genes and 6 down-regulated genes in the group of the single inoculation of *L. paracasei* and 5 up-regulated genes and 6 down-regulated genes in the group of the mixed inoculation. The comparison results of different inoculation methods confirmed six up-regulated genes and three down-regulated genes on the first day of fermentation and three up-regulated genes and three down-regulated genes on the third day of fermentation. The up-regulated genes exhibited positive effects on hexosaminidase (LSEI_0291), PTS system, mannose-specific components (LSEI_0401 and LSEI_0561), phosphoglucomutase (LSEI_0949), glutamine-fructose-6-phosphate transaminase (LSEI_1019), UTP–glucose-1-phosphate uridylyltransferase (LSEI_1093), and glucosamine-6-phosphate deaminase (LSEI_2889).

We further confirmed 17 key DEGs at corresponding positions of starch and sucrose metabolism. The comparison results of different fermentation times (3 and 1 days) confirmed 11 up-regulated genes and three down-regulated genes in the group of the single inoculation of *L. paracasei* and six up-regulated genes and two down-regulated genes in the group of mixed inoculation. The comparison results of different inoculation methods (single inoculation of *L. paracasei* and mixed inoculation) confirmed six up-regulated genes and two down-regulated genes on the first day of fermentation and three up-regulated genes and four down-regulated genes on the third day of fermentation (Supplementary Table 4). The up-regulated genes including oligo-1,6-glucosidase (LSEI_0406), trehalose-6-phosphate hydrolase (LSEI_0630), beta-glucosidase (LSEI_0700), and phosphoglucomutase (LSEI_0949) exhibited positive effects on the fermentation process in the groups of single inoculation of *L. paracasei* and mixed inoculation.

#### Genes Involved in Glycolysis

**FIGURE 5:**
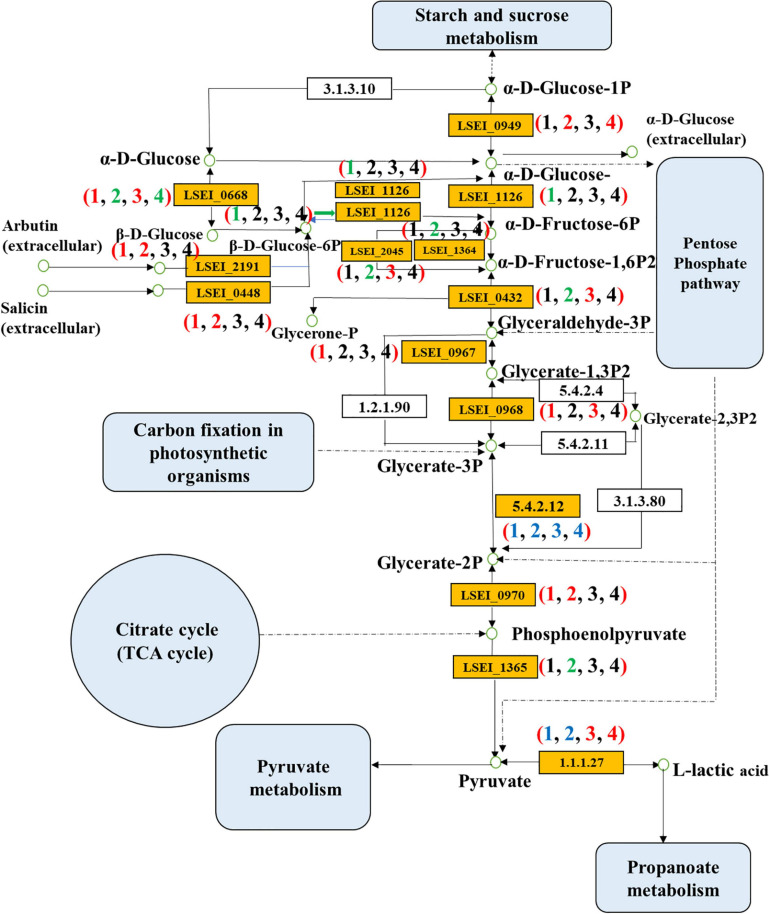
Differentially expressed genes of glycolysis pathway in four pairwise comparisons (Numbers in brackets 1, 2, 3, and 4 refer to the L3 vs L1 days, LY3 vs LY1 days, LY1 vs L1 days, and LY3 vs L3 days, respectively. L1 day refers to single inoculation with *L. paracasei* on the first day, L3 day refers to single inoculation with *L. paracasei* on the third day, LY1 day refers to mix inoculation with *L. paracasei* and *K. marxianus* on the first day, LY3 day refers to mix inoculation with *L. paracasei* and *K. marxianus* on the third day. The colors red, green, blue, and black represent the up-regulated gene, down-regulated gene, both of up-regulated gene and down-regulated gene, and no change gene, respectively).

Different inoculation methods had different enhancement effects on rice-acid metabolism. We further confirmed 20 key DEGs at corresponding positions of glycolysis metabolism (Table 1). The up-regulated and down-regulated genes in glycolysis metabolism were related to carbohydrate transport and metabolism and energy production and conversion (Figure 5). The comparison results of different fermentation times (3 and 1 days) confirmed 10 up-regulated genes and 3 down-regulated genes in the group of single inoculation of *L. paracasei* and 4 up-regulated genes and 8 down-regulated genes in the group of mixed inoculation. The comparison results of inoculation methods (single inoculation of *L. paracasei* and mixed inoculation) confirmed 8 up-regulated genes on the first day of fermentation and 3 up-regulated genes and 2 down-regulated genes on the third day of fermentation. The key genes encoding L-lactate dehydrogenase (LSEI_2549, FC = 1.89), glyceraldehyde 3-phosphate dehydrogenase (LSEI_0967, FC = 1.71), phosphoglycerate kinase (LSEI_0968, FC = 2.22) and enolase (LSEI_0970, FC = 1.59) were up-regulated in the first pairwise comparison (L3 vs L1 days), indicating that these genes could affect the increase of L-lactic acid, sourness and richness in the fermentation process of rice-acid only inoculated with *L. paracasei.* The key genes encoding 6-phosphofructokinase (LSEI_1364, FC = 1.83), fructose-1,6-bisphosphatase III (LSEI_2045, FC = 1.57), fructose-bisphosphate aldolase Class II (LSEI_0432, FC = 1.97), pyruvate kinase (LSEI_1365, FC = 2.00) and l-lactate dehydrogenase (LSEI_2607, FC = 1.95) were down-regulated in the third pairwise comparison (LY3 vs LY1 days), indicating that these genes could have a negative effect on the increase of L-lactic acid, sourness and richness in the fermentation process of rice-acid with mixed inoculation. The comparison results of different inoculation methods in the first day of fermentation indicated that the key genes of LSEI_2549 (FC = 1.61), LSEI_2045 (FC = 2.05), LSEI_0432 (FC = 2.71), and LSEI_0968 (FC = 1.66) exhibited positive effects on the formation of L-lactic acid, whereas the key genes showed no negative effect on the formation of L-lactic acid in the third day of fermentation.

**TABLE 1:** The key genes involved in glycolysis, pyruvate metabolism, and TCA cycle in single inoculation with *L. paracasei* and mix inoculation during rice-acid fermentation process among four pairwise comparisons.

Gene ID	EC ID	KEGG annotation	FC (L3 vs L1 days)	FC (LY3 vs LY1 days)	FC (LY1 vs L1 days)	FC (LY3 vs L3 days)
**Glycolysis**						
LSEI_0331	K15634	Integral membrane protein	4.82	−2.43	5.58	−2.04
LSEI_0432	[EC:4.1.2.13]	K01624 fructose-bisphosphate aldolase, class II	–	−1.97	2.71	–
LSEI_0448	[EC:3.2.1.86]	K01223 6-phospho-beta-glucosidase	2.25	–	–	–
LSEI_0634	[EC:1.1.1.27]	K00016 l-lactate dehydrogenase	–	1.99	–	1.53
LSEI_0668	[EC:5.1.3.3]	Aldose 1-epimerase; K01785 aldose 1-epimerase	–	−3.48	2.81	−1.68
LSEI_0775	[EC:1.1.1.1]	K04072 acetaldehyde dehydrogenase / alcohol dehydrogenase	2.07	–	4.35	1.99
LSEI_0949	[EC:5.4.2.2]	K01835 phosphoglucomutase	–	2.01	–	1.59
LSEI_0967	[EC:1.2.1.12]	K00134 glyceraldehyde 3-phosphate dehydrogenase	1.71	–	–	–
LSEI_0968	[EC:2.7.2.3]	K00927 phosphoglycerate kinase	2.22	–	1.66	–
LSEI_0970	[EC:4.2.1.11]	K01689 enolase	1.59	1.62	–	–
LSEI_1126	[EC:5.3.1.9]	K01810 glucose-6-phosphate isomerase	−1.56	–	–	–
LSEI_1310	[EC:1.1.1.27]	K00016 l-lactate dehydrogenase	−2.17	−2.64	–	–
LSEI_1364	[EC:2.7.1.11]	K00850 6-phosphofructokinase 1	–	−1.83	–	–
LSEI_1365	[EC:2.7.1.40]	K00873 pyruvate kinase	–	−2.00	–	–
LSEI_1446	[EC:1.8.1.4]	K00382 dihydrolipoamide dehydrogenase	2.46	–	1.88	–
LSEI_2045	[EC:3.1.3.11]	K04041 fructose-1,6-bisphosphatase III	–	−1.57	2.04	–
LSEI_2191	[EC:3.2.1.86]	K01223 6-phospho-beta-glucosidase	2.66	2.35	–	–
LSEI_2549	[EC:1.1.1.27]	K00016 l-lactate dehydrogenase	1.89	–	1.61	–
LSEI_2598	[EC:5.1.3.3]	K01785 aldose 1-epimerase	1.58	–	–	–
LSEI_2607	[EC:1.1.1.27]	K00016 l-lactate dehydrogenase	−3.34	1.95	–	–
**Pyruvate metabolism**						
LSEI_0166	[EC:2.7.2.1]	K00925 acetate kinase	–	1.72	–	–
LSEI_0322	[EC:1.3.5.4]	K00244 fumarate reductase flavoprotein subunit	–	–	2.17	–
LSEI_0594	[EC:1.3.5.4]	General function prediction only K00244 fumarate reductase flavoprotein subunit	−1.71	–	–	–
LSEI_0634	[EC:1.1.1.27]	K00016 l-lactate dehydrogenase	–	1.99	–	1.53
LSEI_0775	[EC:1.1.1.1]	Energy production and conversion K04072 acetaldehyde dehydrogenase/alcohol dehydrogenase	2.07	–	4.35	2.00
LSEI_1305	[EC:1.2.4.1]	K00161 pyruvate dehydrogenase E1 component alpha subunit	−1.82	–	–	–
LSEI_1306	[EC:1.2.4.1]	K00162 pyruvate dehydrogenase E1 component beta subunit	−1.83	–	−1.60	–
LSEI_1307	[EC:2.3.1.12]	K00627 pyruvate dehydrogenase E2 component (dihydrolipoamide acetyltransferase)	−1.95	–	−1.96	–
LSEI_1308	[EC:1.8.1.4]	K00382 dihydrolipoamide dehydrogenase	−1.54	–	−1.80	–
LSEI_1310	[EC:1.1.1.27]	K00016 l-lactate dehydrogenase	−2.17	−2.64	–	–
LSEI_1446	[EC:1.8.1.4]	K00382 dihydrolipoamide dehydrogenase	2.46	–	1.88	–
LSEI_1784	[EC:1.2.3.3]	K00158 pyruvate oxidase	3.58	6.73	−1.54	–
LSEI_1787	[EC:2.3.1.9]	Lipid transport and metabolism K00626 acetyl-CoA C-acetyltransferase	−2.30	−2.35	–	–
LSEI_2110	[EC:2.1.3.15]	K01963 acetyl-CoA carboxylase carboxyl transferase subunit beta	1.82	4.38	–	1.75
LSEI_2156	[EC:1.1.1.28]	K03778 d-lactate dehydrogenase	−4.99	–	−4.63	–
LSEI_2172	[EC:2.7.2.1]	K00925 acetate kinase	2.57	3.73	1.64	2.19
LSEI_2410	[EC:4.2.1.2]	K01679 fumarate hydratase, class II	−1.69	−2.00		–
LSEI_2549	[EC:1.1.1.27]	l-lactate dehydrogenase	1.89	–	1.61	–
LSEI_2607	[EC:1.1.1.27]	K00016 l-lactate dehydrogenase	−3.34	−1.95	–	–
LSEI_2866	[EC:1.1.1.38]	K00027 malate dehydrogenase (oxaloacetate-decarboxylating)	3.94	8.46	–	–
**TCA cycle**						
LSEI_0322	[EC:1.3.5.4]	K00244 fumarate reductase flavoprotein subunit	–	–	2.17	–
LSEI_0594	[EC:1.3.5.4]	General function prediction only/K00244 fumarate reductase flavoprotein subunit	−1.71	–	–	–
LSEI_1305	[EC:1.2.4.1]	Energy production and conversion K00161 pyruvate dehydrogenase E1 component alpha subunit	−1.83	–	–	–
LSEI_1306	[EC:1.2.4.1]	K00162 pyruvate dehydrogenase E1 component beta subunit	−1.83	–	−1.60	–
LSEI_1307	[EC:2.3.1.12]	K00627 pyruvate dehydrogenase E2 component (dihydrolipoamide acetyltransferase)	−1.95	–	−1.96	–
LSEI_1308	[EC:1.8.1.4]	K00382 dihydrolipoamide dehydrogenase	−1.54	–	−1.80	–
LSEI_1315	[EC:6.4.1.1]	K01958 pyruvate carboxylase	–	−2.75	1.66	–
LSEI_1446	[EC:1.8.1.4]	K00382 dihydrolipoamide dehydrogenase	2.46	–	1.88	–
LSEI_1820	[EC:4.1.1.49]	K01610 phosphoenolpyruvate carboxykinase (ATP)	–	–	−1.83	–
LSEI_2410	[EC:4.2.1.2]	K01679 fumarate hydratase, class II	−1.69	−2.00	–	–

*– means no significant difference.*

#### Genes Involved in Pyruvate Metabolism

**FIGURE 6:**
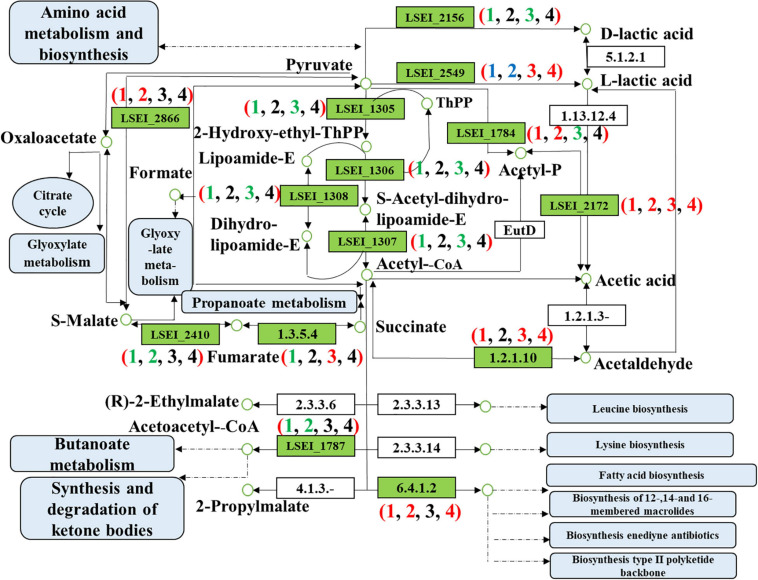
Differentially expressed genes of pyruvate metabolism in four pairwise comparisons (Numbers in brackets 1, 2, 3, and 4 refer to the L3 vs L1 days, LY3 vs LY1 days, LY1 vs L1 days, and LY3 vs L3 days, respectively. L1 day refers to single inoculation with *L. paracasei* on the first day, L3 day refers to single inoculation with *L. paracasei* on the third day, LY1 day refers to mix inoculation with *L. paracasei* and *K. marxianus* on the first day, LY3 day refers to mix inoculation with *L. paracasei* and *K. marxianus* on the third day. The colors red, green, blue, and black represent the up-regulated gene, down-regulated gene, both of up-regulated gene and down-regulated gene, and no change gene, respectively).

The key DEGs involved in pyruvate metabolism were related to energy production and conversion, carbohydrate transport and metabolism, and lipid transport and metabolism. We further confirmed 20 key DEGs at corresponding positions of pyruvate metabolism (Table 1 and Figure 6). The comparison results of different fermentation times (3 and 1 days) confirmed 7 up-regulated genes and 10 down-regulated genes in the group of single inoculation of *L. paracasei* and 6 up-regulated genes and 4 down-regulated genes in the group of mixed inoculation. The comparison results of different inoculation methods confirmed five up-regulated genes and five down-regulated genes on the first day of fermentation and four up-regulated genes on the third day of fermentation. The gene encoding d-lactate dehydrogenase (LSEI_2156, FC = 4.99) in the third day of fermentation showed the lower mRNA level than that on the first day in the group of the single inoculation of *L. paracasei*. Similarly, the gene encoding d-lactate dehydrogenase (LSEI_2156) in the first day of fermentation in the group of the mixed inoculation showed lower mRNA levels than that in the group of the single inoculation of *L. paracasei*. This result reasonably interpreted the phenomenon that the decrease of d-lactate dehydrogenase led to the increase in L-lactic acid. The gene encoding l-lactate dehydrogenase (LSEI_2549, FC = 1.89) in the third day of fermentation showed a higher mRNA level than that on the first day in the group of the single inoculation of *L. paracasei*. Similarly, the gene encoding l-lactate dehydrogenase (LSEI_2549) in the first day of fermentation in the group of the mixed inoculation showed a higher mRNA level than that in the group of the single inoculation of *L. paracasei*. Another gene encoding L-lactate dehydrogenase (LSEI_0634) showed a positive effect on L-lactic acid formation in the two pairwise comparisons (LY3 vs LY1 days; LY3 vs L3 days). These results reasonably demonstrated the increase in the L-lactic acid in the fermentation process in the study. The genes encoding acetate kinase (LSEI_0166 and LSEI_2172) were up-regulated in the four pairwise comparisons and the gene LSEI_2172 had different FC values (2.57, 3.73, 1.64, and 2.19) in the four pairwise comparisons. These data proved that acetic acid showed different increasing trends in the group of mixed inoculation and the group of single inoculation in the rice-acid fermentation process. The up-regulated gene encoding malate dehydrogenase (LSEI_2866, FC = 3.94 and 8.46) reasonably interpreted the increase in malic acid in the fermentation process. The function of up-regulated genes was related to the sourness and bitterness of taste substances measured with E-tongues. The indication requires further experimental verification in the future.

#### Genes Involved in TCA Cycle

The key DEGs in TCA cycle were related to energy production and conversion and general function prediction. We further confirmed 10 DEGs at corresponding positions of TCA cycle (Table 1 and Supplementary Figure 5). The comparison results of different fermentation times (3 and 1 days) confirmed one up-regulated gene and six down-regulated genes in the group with single inoculation of *L. paracasei* and two down-regulated genes in the group with mixed inoculation. The comparison results of different inoculation methods (mixed inoculation and single inoculation of *L. paracasei*) confirmed three up-regulated genes and four down-regulated genes on the first day of fermentation, but no up-regulated or down-regulated genes were confirmed on the third day of fermentation. The gene encoding dihydrolipoamide dehydrogenase (LSEI_1446, FC = 2.46) in the third day showed a higher mRNA level than that on the first day in the group with single inoculation of *L. paracasei*. Although six genes were down-regulated, their FC values were low. The comparison results of two inoculation methods indicated that the genes encoding fumarate reductase flavoprotein subunit (LSEI_0322, FC = 2.17), pyruvate carboxylase (LSEI_1315, FC = 1.66), and dihydrolipoamide dehydrogenase (LSEI_1446, FC = 1.88) showed higher mRNA levels on the first day of fermentation.

#### Genes Involved in Amino Acid Biosynthesis and Metabolism

In order to identify the differences in main amino acid metabolic pathways between single inoculation and mixed inoculation, we further confirmed DEGs and analyzed 10 amino acid metabolic pathways (Supplementary Table 5). The comparison results of different fermentation time (3 and 1 days) indicated that the genes of arginine and proline metabolism (*P* = 0.04), arginine biosynthesis (*P* = 0.05), and histidine metabolism (*P* = 0.02) showed the significant difference in the group with single inoculation of *L. paracasei*, whereas the genes of cysteine and methionine metabolism (*P* = 0.006) and lysine biosynthesis (*P* = 0.004) showed the extremely significant difference in the group with mixed inoculation. The comparison results of two inoculation methods indicated that the genes of alanine, aspartate, and glutamate metabolism (*P* = 0.003) showed the extremely significant difference in the first day of fermentation, whereas the genes of histidine metabolism (*P* = 0.0001) and tyrosine metabolism (*P* = 0.0379), respectively, showed the extremely significant difference and significant difference in the third day of fermentation.

## Discussion

Rice-acid as a functional fermented product is favored by Chinese consumers. Due to its rich probiotic flora, unique flavor, and nutrients, rice-acid is being developing into a beverage. Since the majority of fermentation processes rely on the conversion of sugars to lactic acid, LAB plays the most important role, followed by yeasts (Tofalo et al., 2019). Notably, during the production process, rice-acid undergoes a series of flavor changes. In order to investigate the molecular mechanisms of formation and metabolic pathways of flavors in rice-acid fermented with *L. paracasei* under the conditions of different fermentation time and inoculation methods. In this study, we investigated the transcriptome of *L. paracasei* during the maturation of rice-acid. Firstly, we measured the cell densities of *L. paracasei* and *K. marxianus* during the different fermentation times. Then, we obtained the key organic acids and taste substances of different fermented rice-acids. Finally, we compared the DEGs related to the KEGG pathways based on comparative transcriptomics.

The cell density of *K. marxianus* in the group of mixed inoculation was lower than that in the group of single inoculation because *K. marxianus* was more adapted to the rice-acid fermentation system and might compete with *L. paracasei* in the late fermentation stage (the data were not shown). *L. paracasei* and *K. marxianus* could cooperate or compete with each other and contribute to the production of some important flavors in rice-acid due to the increased flux in the acetyl-CoA synthetic pathway and TCA cycle of *K. marxianus* (Sakihama et al., 2019). The metabolism pathway of *L. paracasei* was discussed in the study. It is worth noting that *L. paracasei* has special probiotic functions. A recent report demonstrated that the pomegranate beverages with the better quality could be obtained by the inoculation with *L. paracasei* (Mantzourani et al., 2020). Interestingly, L-lactic acid was the most important organic acid in rice-acid because the inoculation with *L. paracasei* could produce L-lactic acid (Thakur et al., 2019). It could be inferred that *L. paracasei* H4-11 played an important role in the formation of L-lactic acid. In addition, L-lactic acid could form poly-L-lactic acid, which could be combined with functional additives to improve their mechanical and biological properties for cardiovascular implantation applications (Kang et al., 2019). Therefore, it is necessary to develop fermented rice-acid with the L-lactic acid metabolism ability. Among eight taste substances in the group of mixed inoculation, the sourness had the highest intensity due to the dynamics from saccharification to alcoholization and acidification in the fermentation process of rice-acid inoculated with lactobacillus and yeasts (Jiao et al., 2017). The result also demonstrated that mixed inoculation had a synergistic effect on the acid production capacity. The variation in sourness was consistent with the variation in L-lactic acid in this study.

*R*-values proved that our experimental results obtained with comparative transcriptomics had high biological repeatability. In addition, different *R*-values in the pairwise comparisons proved the rationality of the experimental design. The four pairwise comparisons suggested different KEGG metabolism pathways, thus resulting in the different concentrations of L-lactic acid, other organic acids, and taste substances. The enrichment of the differentially transcribed genes in the categories of beta-Lactam resistance and photosynthesis was obtained in three pairwise comparisons except for the third pairwise comparison (LY1 vs L1 days). Importantly, the KEGG metabolism pathways of starch and sucrose metabolism, glycolysis/gluconeogenesis, pyruvate metabolism, TCA cycle, and amino acid biosynthesis and metabolism showed the differences in the four pairwise comparisons due to the difference in the sourness and taste characteristics of different rice-acid samples. It is worth noting that the enrichment of the differentially transcribed genes in glutathione metabolism in the first pairwise comparison was higher than that in the other three pairwise comparisons. Glutathione S-transferase (LSEI_1467) and glutathione peroxidase (LSEI_0893) found in *L. paracasei* were reported to play an important role in acid stress response in other LAB (Lee et al., 2010). Interestingly, we found that the differentially transcribed genes were enriched in the longevity regulation pathway, thus interpreting the longevity of local residents who often drank rice-acid. The indication requires to be further experimentally verified.

Most ribosomal genes were related to the functions of translation, ribosomal structure, and biogenesis in this study. The down-regulated genes were related to the ribosome, indicating that total ribosomes limited protein synthesis and growth, as reported by Zhang et al. (2020). In addition, the up-regulated genes indicated that the synthesis pathway of ribosomes provided more energy and resources for other cellular components. Most ABC transporters were related to the functions of amino acid transport and metabolism, energy production and conversion, inorganic ion transport and metabolism, and defense mechanisms. Most genes showed a lower mRNA level on the third day because the third day was the end of fermentation. The result also confirmed our proper choice of fermentation time. Purine metabolism refers to the metabolic pathways that synthesize and degrade purines in many organisms (Klotz et al., 2020). Most purine metabolism-related genes were related to the functions of nucleotide transport and metabolism, carbohydrate transport and metabolism, general function prediction, replication, recombination, repair, and transcription. The down-regulated genes prevented the purine nucleotide synthesis, thus leading to significant phosphate consumption in yeasts (Pinson et al., 2009). Up-regulated and down-regulated genes strongly confirmed the interactive symbiotic system of *L. paracasei* and *K. marxianus*.

Amino sugar and nucleotide sugar were the important precursors of flavor substances in the fermentation system of rice-acid. The abundance of amino sugar and nucleotide sugar could affect the glycan complexity and the final glycosylation profile (Naik et al., 2018). The comparison results of different inoculation methods indicated that the down-regulated genes were less than the up-regulated genes. This result suggested that these up-regulated genes promoted the fermentation process of rice-acid in the interaction between *L. paracasei* H4-11 and *K. marxianus* L1-1. In addition, a recent study demonstrated that amino sugar and nucleotide sugar metabolism played the important role in the interactions between *L. sanfranciscensis* and *S. cerevisiae* (Yang et al., 2020). The demonstration was consistent with the interactions between *L. paracasei* and *K. marxianus* in our study.

Starch and sucrose were also the important precursors of flavor substances in the fermentation system of rice-acid. Under the action of enzymes, starch and sucrose were hydrolyzed and converted into acids and other taste components (Li et al., 2020). Interestingly, the comparison results of different inoculation methods indicated that the gene encoding UTP–glucose-1-phosphate uridylyltransferase (LSEI_1093) was up-regulated. This gene (LSEI_1093) played a crucial role in the mixed inoculation. The genes encoding alpha-glucosidase (LSEI_0980 and LSEI_2102), maltose phosphorylase (LSEI_0982), and beta-phosphoglucomutase (LSEI_0983) in the group of the mixed inoculation showed lower mRNA levels than those in the group of the single inoculation, indicating that the three genes exhibited negative effects on the mixed inoculation in rice-acid. Those genes might be associated with the decrease in bitterness intensity and astringency intensity in the group of mixed inoculation compared to the group of single inoculation of *L. paracasei*.

Glycolysis is a central and important metabolic pathway and can provide energy, reducing power, and pyruvate to fuel the TCA cycle, and precursors for amino acid, fatty acid, and secondary metabolite biosynthesis (Plaxton, 1996). The key glycolysis genes discussed in the study included up-regulated and down-regulated genes during the fermentation period from the organic acid formation stage to the taste substance maturation and ripening stages. Different gene states resulted in different taste substances in rice-acid with single inoculation or mixed inoculation during the fermentation process. The up-regulation of glycolytic genes in the exponential phase could provide the energy for the rapid growth of strains (Qiao et al., 2019). Glycolysis is the important metabolic pathway, which is related to the formation of L-lactic acid. Up-regulation of L-lactate dehydrogenase indicated that L-lactic acid was mainly produced in the early fermentation phase for energy production. The down-regulation of the genes encoding pyruvate kinase (LSEI_1365) and 6-phosphofructokinase (LSEI_1364) indicated that L-lactic acid was stable in the late fermentation stage. The different results for LSEI_2549 could be caused by the post-transcriptional regulation. Consistently, the genes encoding l-lactate dehyrogenase (LSEI_2549) and 6-phosphofructokinase (LSEI_1364) had been identified to promote bacterial adhesion to mucin and epithelial cells (Izquierdo et al., 2009). We could infer that mixed inoculation with *L. paracasei* H4-11 and *K. marxianus* L1-1 had an interactive symbiotic relationship, as reported in the previous study (Ishii et al., 1999). Therefore, the key genes could affect the content of L-lactic acid, sourness, and richness in the fermentation process of rice-acid.

The pyruvate metabolism contributes to the production of α-keto acids, which is the prerequisite for the synthesis of more flavor substances in yeasts (Sun et al., 2019). Yeasts play an important role in rice-acid fermentation. Therefore, *L. paracasei* H4-11 and *K. marxianus* L1-1 strains were inoculated in rice-acid. In our study, the genes encoding acetate kinase (LSEI_0166), pyruvate oxidase (LSEI_1784), L-lactate dehyrogenase (LSEI_2549), and malate dehydrogenase (LSEI_2866) showed the up-regulation trend. A recent report proved that the genes (L-lactate dehydrogenase and pyruvate kinase) had been identified to promote L-lactic acid formation (Yao et al., 2019), so the DEGs in pyruvate metabolism strongly proved that *L. paracasei* and *K. marxianus* had a symbiotic relationship. Thus, the metabolic pathways of pyruvate were also the decisive factor in the formation of flavor compounds in the group of mixed inoculation. Interestingly, the previous study proved that in addition to the transformation from EMP to PKP, more redox-related metabolic reactions were affected by the co-cultivation of LAB and yeasts which were involved in pyruvate metabolism (Xu et al., 2019). We will further explore the antioxidant mechanism of rice-acid with mixed inoculation.

TCA cycle is a series of chemical reactions for the oxidation of acetyl-CoA derived from carbohydrates, fats, and proteins to provide energy. TCA cycle-related metabolites (malic acid and other organic acids) act as energy storage materials during the fermentation process of rice-acid inoculated with *L. paracasei*. Functionally, carbohydrate metabolism influences a number of processes including glycolysis, TCA cycle, and hexose metabolism, and may be associated with the expansion of *L. paracasei* in rice-acid fermentation as well as the generation of energy (Mendes Ferreira and Mendes-Faia, 2020). Furthermore, dihydrolipoamide dehydrogenase (LSEI_1446) is a crucial enzyme in the fermentation process and may be related to the formation of taste substances. Notably, dihydrolipoamide dehydrogenase (LSEI_1446) is related to the antioxidant function and it is a redox enzyme involved in decarboxylation of pyruvate to form acetyl-CoA in the cascade of glucose metabolism and mitochondrial adenine triphosphate (ATP) production (Yang et al., 2019). The genes involved in the TCA cycle and related to amino acids (the precursor of taste substances) included LSEI_0322, LSEI_1307, LSEI_1308, and LSEI_1820 and showed different expression in the four comparisons. The result indicated that mixed inoculation had a significant effect on the TCA cycle in *L. paracasei*. The function of up-regulated genes might be responsible for the differences in taste substances, including the substances of sourness, and other taste characteristics measured with E-tongues.

The amino acid metabolism pathways showed the close relationship with taste substances in fermented foods (Yu et al., 2015; Zhao et al., 2016; Hu et al., 2020). Arginine and proline metabolism, arginine biosynthesis, and tyrosine metabolism had a positive effect on the formation of umami and bitterness. Histidine metabolism had a positive effect on the formation of saltiness. Cysteine and methionine metabolism had a positive effect on the formation of astringency and saltiness. Lysine biosynthesis had a positive effect on bitterness and richness in the fermentation process. Alanine, aspartate, and glutamate metabolism in the group of mixed inoculation led to a higher sourness value than that in the group of single inoculation. Alanine, aspartate, and glutamate metabolites might be the precursors of L-lactic acid. These pathways verified the results of taste substances measured with E-tongue. The different pathways contributed to taste substances generated in the rice-acid fermentation process in the group of single inoculation of *L. paracasei* and the group of mixed inoculation. Furthermore, most amino acids promoted the formation of bitterness. The structural requirements for ACE-inhibitory activity were related to the structural characteristics of bitter peptides and many bitter dipeptides showed the ACE-inhibitory activity (Pripp and Ardö, 2007). These results of amino acid metabolism reasonably demonstrated the positive effects of the symbiotic system composed of two strains (*L. paracasei* H4-11 and *K. marxianus* L1-1) on the formation of taste characteristics in rice-acid.

The genes related to the metabolism of some amino acids showed higher relative mRNA levels, including alanine, aspartate, glutamate, arginine, and proline metabolism, which had a crucial effect on the formation of taste substances. Especially, the genes encoding fumarate reductase flavoprotein subunit (LSEI_0322), alcohol dehydrogenase (LSEI_0775), and glycine ligase (LSEI_1746) showed a significant and positive correlation with the umami intensity. The genes encoding the PTS system, mannose-specific IIB component (LSEI_0401), and large subunit ribosomal protein L7/L12 (LSEI_2272) showed the significant and negative correlation with astringency intensity. The gene encoding L-lactate dehyrogenase (LSEI_2549) showed the significant and negative correlation with bitterness intensity, indicating the correlation with the formation of amino acids. Moreover, the produced amino acids were converted into corresponding α-keto acids by aminotransferases. The conversion of amino acids to α-keto acids is the first step in the formation of taste substances (Pangallo et al., 2019). Branched-chain amino acids are crucial precursors of protein synthesis. Leucine plays an important role in the regulation of intracellular signal transduction for the control of mRNA translation (Liu et al., 2014). Transamination of leucine is associated with the production of 3-methylbutanoic acid, and serine deamination may supply pyruvate, which can be subsequently converted into acetic acid, 2,3-butanedione, and acetoin (Wang et al., 2020). Amino acid metabolism is not only the starting compound for the synthesis of various metabolites such as glycine, cysteine, and serine phospholipids but also building blocks for protein synthesis (Shimizu et al., 2008). Moreover, most amino acids are crucial metabolites that play an important role in the growth and protein synthesis of *L. paracasei* in the rice-acid fermentation process.

## Conclusion

In summary, the study provides a comprehensive overview of the global changes at the transcription level in the growth of *L. paracasei* in the rice-acid fermentation process. Most of the observed growth phase-dependent changes at the mRNA level appeared in the early stage and the mRNA levels of relevant genes became stable in the later stage. In particular, the genes related to amino sugar and nucleotide sugar metabolism and starch and sucrose metabolism affected the energy required for the growth of *L. paracasei* in the early stage. Many transcripts related to glycolysis, pyruvate metabolism, TCA cycle, and amino acid biosynthesis and metabolism were differentially expressed in the growth of *L. paracasei* in the presence of *K. marxianus*. The differential expressions of the genes related to the synthesis of L-lactic acid and other organic acids and taste characteristics were observed in the whole growth period. The DEGs of various metabolic pathways revealed the formation mechanism of L-lactic acid. The study provides the theoretical basis for the industrial applications of the symbiotic system of LAB and yeasts.

## Data Availability Statement

The datasets presented in this study can be found in online repositories. The names of the repository/repositories and accession number(s) can be found below: BioProject accession PRJNA658256.

## Author Contributions

NL performed the experiments, analyzed the data, and prepared the manuscript. LQ and SM contributed to the experimental design, manuscript revision, and overall support of this study. All authors read and approved the final manuscript.

## Conflict of Interest

The authors declare that the research was conducted in the absence of any commercial or financial relationships that could be construed as a potential conflict of interest.

## Footnotes

1 http://bacteria.ensembl.org/Lactobacillus_paracasei_atcc_334_gca_000014525/Info/Index
