# Supplementary material for: Regulatory Mechanisms of L-Lactic Acid and Taste Substances in Chinese Acid Rice Soup (Rice-Acid) Fermented With a Lacticaseibacillus paracasei and Kluyveromyces marxianus
Source: Front Microbiol. 2021 May 21;12:594631. doi: 10.3389/fmicb.2021.594631 (PMC8176858; doi:10.3389/fmicb.2021.594631)
Supplement: Supplementary file 1 [file Data_Sheet_1.docx]

**Supplementary Material**

**Regulatory Mechanisms of L-lactic acid and Taste Substances in Chinese Acid Rice Soup (Rice-acid) fermented with a Novel *Lacticaseibacillus Paracasei* and *Kluyveromyces Marxianus***

**Na Liu^1, 3^, Likang Qin^1,2,*^, Song Miao^3*^**

^1^ Key laboratory of Plant Resource Conservation and Germplasm Innovation in Mountainous Region (Ministry of Education), Collaborative Innovation Center for Mountain Ecology & Agro-Bioengineering (CICMEAB), College of Life Sciences/Institute of Agro-bioengineering, Guizhou University, Guiyang 550025, China.

^2^School of Liquor and Food Engineering, Guizhou University, Guiyang, 550025, China.

^3^Teagasc Food Research Centre, Moorepark, Fermoy, Co. Cork, Ireland.

*** Correspondence:**Likang Qin
lkqin@gzu.edu.cn

Song Miao

song.miao@teagasc.ie

TABLE S1. Quality assessment results of *L. paracasei* RNA sequencing in the sample of L1 d, L3 d, LY1 d and LY3 d.

| Sample ID | Read Sum | Base Sum | GC (%) | Q20 (%) | Q30 (%) |
| --- | --- | --- | --- | --- | --- |
| L1 d-1 | 9,596,269 | 2,878,880,700 | 50.29% | 97.54% | 92.70% |
| L1 d-2 | 9,100,262 | 2,730,078,600 | 48.40% | 97.70% | 93.05% |
| L1 d-3 | 8,840,965 | 2,652,289,500 | 48.26% | 97.66% | 92.96% |
| L3 d-1 | 7,824,981 | 2,347,494,300 | 45.73% | 97.55% | 92.66% |
| L3 d-2 | 8,515,425 | 2,554,627,500 | 45.59% | 97.74% | 93.11% |
| L3 d-3 | 8,248,125 | 2,474,437,500 | 45.40% | 97.73% | 93.03% |
| LY1 d-1 | 8,434,452 | 2,530,335,600 | 45.49% | 97.63% | 92.84% |
| LY1 d-2 | 9,165,968 | 2,749,790,400 | 45.42% | 97.57% | 92.71% |
| LY1 d-3 | 10,228,721 | 3,068,616,300 | 45.25% | 97.59% | 92.71% |
| LY3 d-1 | 9,738,178 | 2,921,453,400 | 45.47% | 97.71% | 93.00% |
| LY3 d-2 | 8,480,934 | 2,544,280,200 | 45.40% | 97.59% | 92.73% |
| LY3 d-3 | 7,989,319 | 2,396,795,700 | 45.73% | 97.77% | 93.16% |

TABLE S2. All DEGs, up-regulated genes and down-regulated genes of *L. paracasei* in four pairwise comparisons.

| DEG Set | All DEG | up-regulated | down-regulated |
| --- | --- | --- | --- |
| L3 d vs L1 d | 1141 | 570 | 571 |
| LY3 d vs LY1 d | 1094 | 517 | 577 |
| LY1 d vs L1 d | 601 | 327 | 274 |
| LY3 d vs L3 d | 329 | 138 | 191 |

TABLE S3. The key genes involved in ribosome, ABC transporters and purine metabolism in single inoculation with *L. paracasei* and mix inoculation during rice-acid fermentation process among four pairwise comparisons.

| Gene ID | KEGG annotation | FC(L3 d vs L1 d) | FC(LY3 d vs LY1 d) | FC(LY1 d vs L1 d) | FC(LY3 d vs L3 d) |
| --- | --- | --- | --- | --- | --- |
| **Ribosome** |  |  |  |  |  |
| LSEI_1358 | K02911 large subunit ribosomal protein L32 | 1.72 | 1.73 | -- | -- |
| LSEI_2272 | K02935 large subunit ribosomal protein L7/L12 | 1.77 | 2.16 | -1.91 | -1.64 |
| LSEI_2476 | K02879 large subunit ribosomal protein L17 |  | -1.69 | -- | -1.71 |
| LSEI_2510 | K02950 small subunit ribosomal protein S12 |  | -2.27 | -- | -1.59 |
| **ABC transporters** |  |  |  |  |  |
| LSEI_0175 | K15580 oligopeptide transport system substrate-binding protein | -4.32 | -3.01 | -2.41 | -1.75 |
| LSEI_0601 | K02424 L-cystine transport system substrate-binding protein | 4.76 | -6.02 | 6.96 | -3.76 |
| LSEI_1177 | K02073 D-methionine transport system substrate-binding protein | 3.73 | -2. 66 | 3.36 | -2.85 |
| LSEI_1592 | K18890 ATP-binding cassette, subfamily B, multidrug efflux pump | -1.61 | -- | -1.93 | -1.57 |
| LSEI_2061 | K10823 oligopeptide transport system ATP-binding protein | -5.43 | -4.03 | -2.00 | -1.55 |
| **Purine metabolism** |  |  |  |  |  |
| LSEI_0122 | K01939 adenylosuccinate synthase [EC:6.3.4.4] | -1.62 | -2.53 | 2.81 | 1.78 |
| LSEI_0949 | K01835 phosphoglucomutase [EC:5.4.2.2] | -- | 2.01 | -- | 1.59 |
| LSEI_1557 | K00759 adenine phosphoribosyltransferase [EC:2.4.2.7] | -- | -2.27 | -- | -1.67 |
| LSEI_1746 | K01945 phosphoribosylamine---glycine ligase [EC:6.3.4.13] | -- | -3.84 | 5.54 | 2.11 |
| LSEI_2229 | putative integral membrane protein with a TlyC-like hemolysin domain | 1.82 | 2.87 | -- | 1.68 |
| LSEI_2287 | K00527 ribonucleoside-triphosphate reductase (thioredoxin) [EC:1.17.4.2] | -- | 2.23 | -- | 2.01 |
| LSEI_2477 | K03040 DNA-directed RNA polymerase subunit alpha [EC:2.7.7.6] | -- | -1.65 | -- | -1.95 |
| LSEI_2515 | K03046 DNA-directed RNA polymerase subunit beta' [EC:2.7.7.6] | -- | -2.55 | -- | -2.03 |

Note: -- means no significant difference.

TABLE S4. The key genes involved in [amino sugar and nucleotide sugar metabolism](file:///D:\E盘\秦老师\白酸汤送样检测结果\转录组学和蛋白组学\转录组学\zx-20191209-578\zx-20191209-578\report\5.DEG_Analysis\L1d-1_L1d-2_L1d-3_vs_L3d-1_L3d-2_L3d-3\pathway\kegg_map\ko00520.html) in single inoculation with *L. paracasei* and mix inoculation during rice-acid fermentation process among four pairwise comparisons.

| Gene ID | EC | Description | FC(L3d vs L1d) | FC(LY3d vs LY1d) | FC(LY1d vs L1d) | FC(LY3d vs L3d) |
| --- | --- | --- | --- | --- | --- | --- |
| [**Amino sugar and nucleotide sugar metabolism**](file:///D:\E盘\秦老师\白酸汤送样检测结果\转录组学和蛋白组学\转录组学\zx-20191209-578\zx-20191209-578\report\5.DEG_Analysis\L1d-1_L1d-2_L1d-3_vs_L3d-1_L3d-2_L3d-3\pathway\kegg_map\ko00520.html) | | | | | | |
| LSEI_0291 | [EC:3.2.1.52] | K12373 hexosaminidase | 2.08 | 1.69 | -- | -- |
| LSEI_0401 | [EC:2.7.1.191] | K02794 PTS system, mannose-specific IIB component | 1.97 | 2.60 | -- | -- |
| LSEI_0405 | [EC:2.7.1.191] | K02793 PTS system, mannose-specific IIA component | 2.06 | -- | -- | -- |
| LSEI_0640 | [EC:5.1.3.2] | K01784 UDP-glucose 4-epimerase | -- | 1.79 | -- | -- |
| LSEI_0664 | [EC:2.7.1.6] | K00849 galactokinase | 1.77 | -5.31 | 2.95 | -3.10 |
| LSEI_0665 | [EC:5.1.3.2] | K01784 UDP-glucose 4-epimerase | -- | -2.81 | 2.07 | -1.65 |
| LSEI_0666 | [EC:2.7.7.12] | K00965 UDPglucose--hexose-1-phosphate uridylyltransferase | 1.58 | -3.07 | 2.64 | -1.77 |
| LSEI_0949 | [EC:5.4.2.2] | K01835 phosphoglucomutase | -- | 2.01 | -- | 1.59 |
| LSEI_1003 | [EC:1.3.1.98] | K00075 UDP-N-acetylmuramate dehydrogenase | 1.55 | -- | -- | -- |
| LSEI_1019 | [EC:2.6.1.16] | K00820 glutamine---fructose-6-phosphate transaminase (isomerizing) | 1.82 | -- | 2.04 | -- |
| LSEI_1022 | [EC:2.7.7.27] | K00975 glucose-1-phosphate adenylyltransferase | 1.52 | -- | -- | -- |
| LSEI_1126 | [EC:5.3.1.9] | K01810 glucose-6-phosphate isomerase | -1.56 | -- | -- | -- |
| LSEI_1093 | [EC:2.7.7.9] | K00963 UTP--glucose-1-phosphate uridylyltransferase | -- | -- | 1.58 | 1.55 |
| LSEI_1808 | [EC:3.5.1.25] | K01443 N-acetylglucosamine-6-phosphate deacetylase | -2.07 | -1.51 | -- | -- |
| LSEI_2018 | [EC:5.1.3.2] | K01784 UDP-glucose 4-epimerase | -3.12 | -4.47 | -- | -- |
| LSEI_2038 | [EC:2.7.7.27] | K00975 glucose-1-phosphate adenylyltransferase | -1.75 | -- | -1.71 | -- |
| LSEI_2039 | [EC:2.7.7.27] | K00975 glucose-1-phosphate adenylyltransferase | -2.33 | -- | -1.58 | -- |
| LSEI_2570 | [EC:2.5.1.7] | K00790 UDP-N-acetylglucosamine 1-carboxyvinyltransferase | 1.64 | -- | -- | -- |
| LSEI_2774 | [EC:4.2.1.126] | K07106 N-acetylmuramic acid 6-phosphate etherase | 2.25 | -1.87 | 2.30 | -- |
| LSEI_2829 | [EC:2.7.1.191] | K02794 PTS system, mannose-specific IIB component | -2.35 | -- | -1.54 | -- |
| LSEI_2889 | [EC:3.5.99.6] | K02564 glucosamine-6-phosphate deaminase | -- | 1.60 | -- | 1.54 |
| **Starch and sucrose metabolism** | | | | | | |
| LSEI_0404 | [EC:3.2.1.20] | K01187 alpha-glucosidase | 1.75 | -- | -- | -- |
| LSEI_0406 | [EC:3.2.1.10] | K01182 oligo-1,6-glucosidase | 2.46 | 1.64 | -- | -- |
| LSEI_0630 | [EC:3.2.1.93] | K01226 trehalose-6-phosphate hydrolase | 1.51 | 2.69 | -- | 1.96 |
| LSEI_0700 | [EC:3.2.1.21] | K05349 beta-glucosidase | 2.04 | 1.82 | 1.54 | -- |
| LSEI_0949 | [EC:5.4.2.2] | K01835 phosphoglucomutase | -- | 2.01 | -- | 1.59 |
| LSEI_0980 | [EC:3.2.1.20] | K01187 alpha-glucosidase | 5.03 | -- | 2.23 | -2.81 |
| LSEI_0982 | [EC:2.4.1.8] | K00691 maltose phosphorylase | 5.06 | -1.72 | 2.81 | -3.10 |
| LSEI_0983 | [EC:5.4.2.6] | K01838 beta-phosphoglucomutase | 3.14 | -1.93 | 2.08 | -2.91 |
| LSEI_1022 | [EC:2.7.7.27] | K00975 glucose-1-phosphate adenylyltransferase | 1.52 | -- | -- | -- |
| LSEI_1093 | [EC:2.7.7.9] | K00963 UTP--glucose-1-phosphate uridylyltransferase | -- | -- | 1.58 | 1.55 |
| LSEI_1126 | [EC:5.3.1.9] | K01810 glucose-6-phosphate isomerase | -1.56 | -- | -- | -- |
| LSEI_2035 | [EC:3.2.1.20] | K01187 alpha-glucosidase | 1.93 | -- | -- | -- |
| LSEI_2038 | [EC:2.7.7.27] | K00975 glucose-1-phosphate adenylyltransferase | -1.75 | -- | -1.71 | -- |
| LSEI_2039 | [EC:2.7.7.27] | K00975 glucose-1-phosphate adenylyltransferase | -2.33 | -- | -1.58 | -- |
| LSEI_2102 | [EC:3.2.1.20] | K01187 alpha-glucosidase | 4.66 | -- | 2.62 | -2.03 |
| LSEI_2104 | [EC:3.2.1.26] | K01193 beta-fructofuranosidase | 2.33 | 2.13 | -- | -- |
| LSEI_2684 | [EC:3.2.1.122] | K01232 maltose-6'-phosphate glucosidase | -- | 2.55 | -- | -- |

Note: -- means no significant difference.

TABLE S5. Amino acid KEGG enrichment of the differentially expressed genes in the four pairwise comparisons.

| Pathway | Amino acid taste^39-45^ | P (L3 d vs L1 d) | P (LY3 d vs LY1 d) | P (LY1 d vs L1 d) | P (LY3 d vs L3 d) |
| --- | --- | --- | --- | --- | --- |
| Alanine, aspartate and glutamate metabolism | Ala (bitterness, sweet, umami), Asp (sourness, umami), Glu(sourness, umami) | ＞0.05 | ＞0.05 | 0.0027 | ＞0.05 |
| Arginine and proline metabolism | Arg (umami, sweet/bitter),  Pro (bitterness) | 0.04 | ＞0.05 | -- | -- |
| Arginine biosynthesis | Arg (umami, sweet/bitter) | 0.05 | ＞0.05 | -- | -- |
| Cysteine and methionine metabolism | Cys(sweetness and saltiness), Met(sweet, astringency) | ＞0.05 | 0.0058 | 0.0995 | 0.0606 |
| Glycine, serine and threonine metabolism | Glycine (sweetness), Ser (sweetness), Thr (saltiness, sweetness) | ＞0.05 | ＞0.05 | ＞0.05 | ＞0.05 |
| Histidine metabolism | His (saltiness, sweetness) | 0.02 | ＞0.05 | -- | 0.0001 |
| Lysine biosynthesis | Lys (bitterness, sweet/bitter) | 0.069 | 0.0037 | ＞0.05 | ＞0.05 |
| Phenylalanine metabolism | Phe (bitterness) | 0.094 | 0.0665 | -- | ＞0.1 |
| Tyrosine metabolism | Tyr (bitterness,umami) | ＞0.05 | ＞0.05 | ＞0.05 | 0.0379 |
| Valine, leucine and isoleucine degradation | Val (bitterness, astringency), Leu (sourness), Iso(bitterness) | ＞0.05 | ＞0.05 | 0.0977 | -- |

Note: -- means no significant difference.


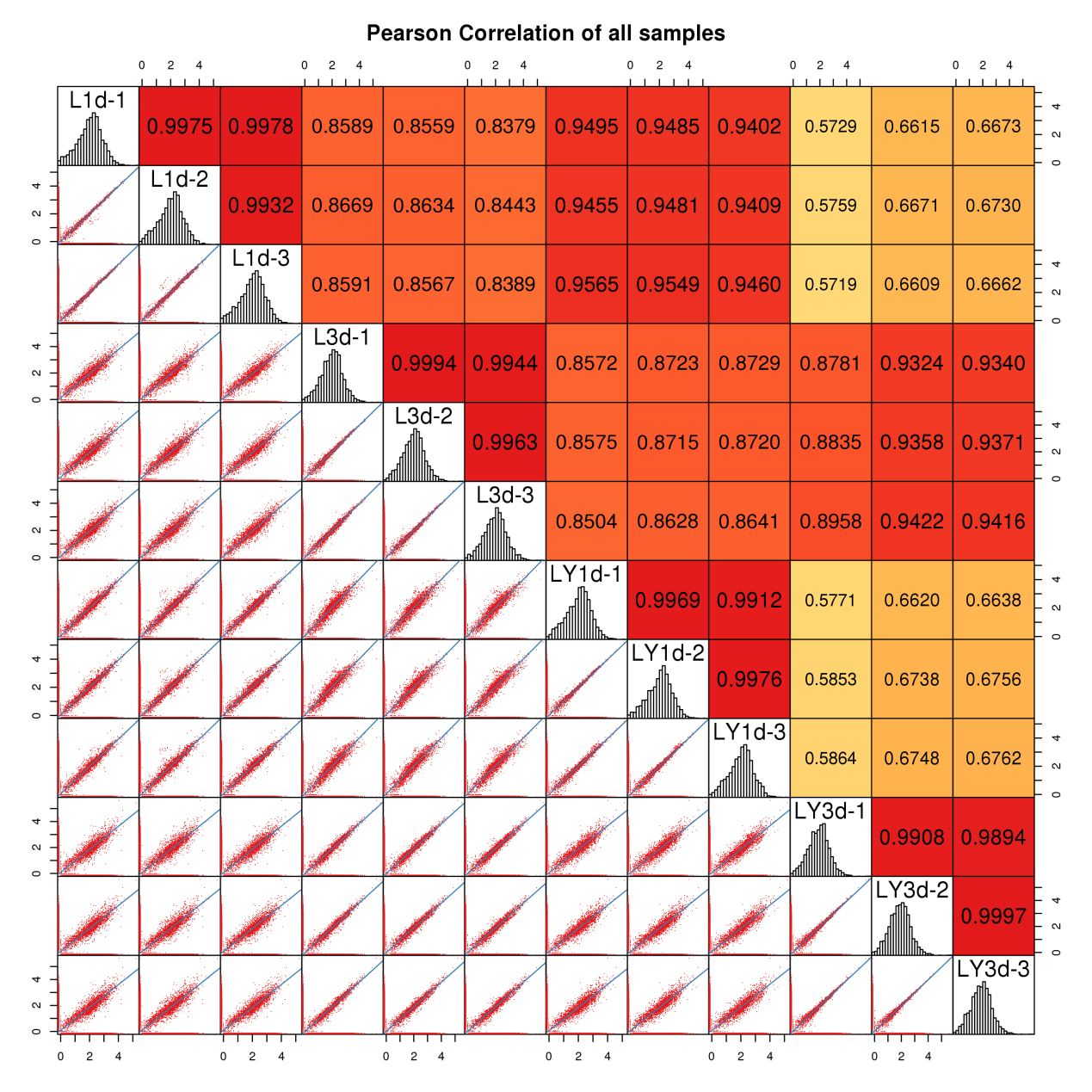


FIGURE S1. Pearson correlation coefficient analysis results of four different experiments inoculated with *L.* *paracasei* with three biological repeats.


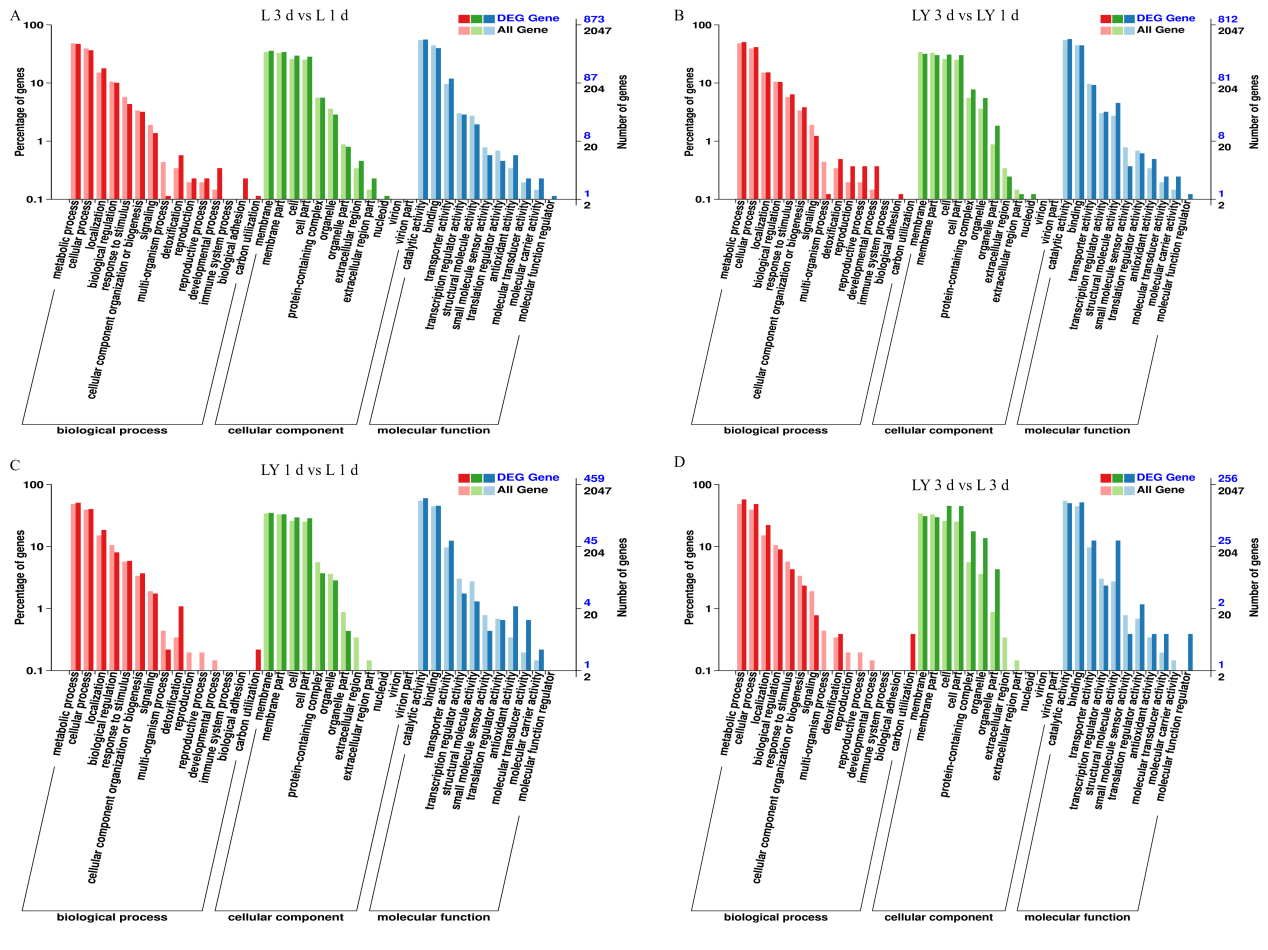


FIGURE S2. Statistical diagram of the second node annotation of the differentially expressed gene GO (A: L3 d vs L1 d, B: LY3 d vs LY1 d, C: LY1 d vs L1 d, D: LY3 d vs L3 d).


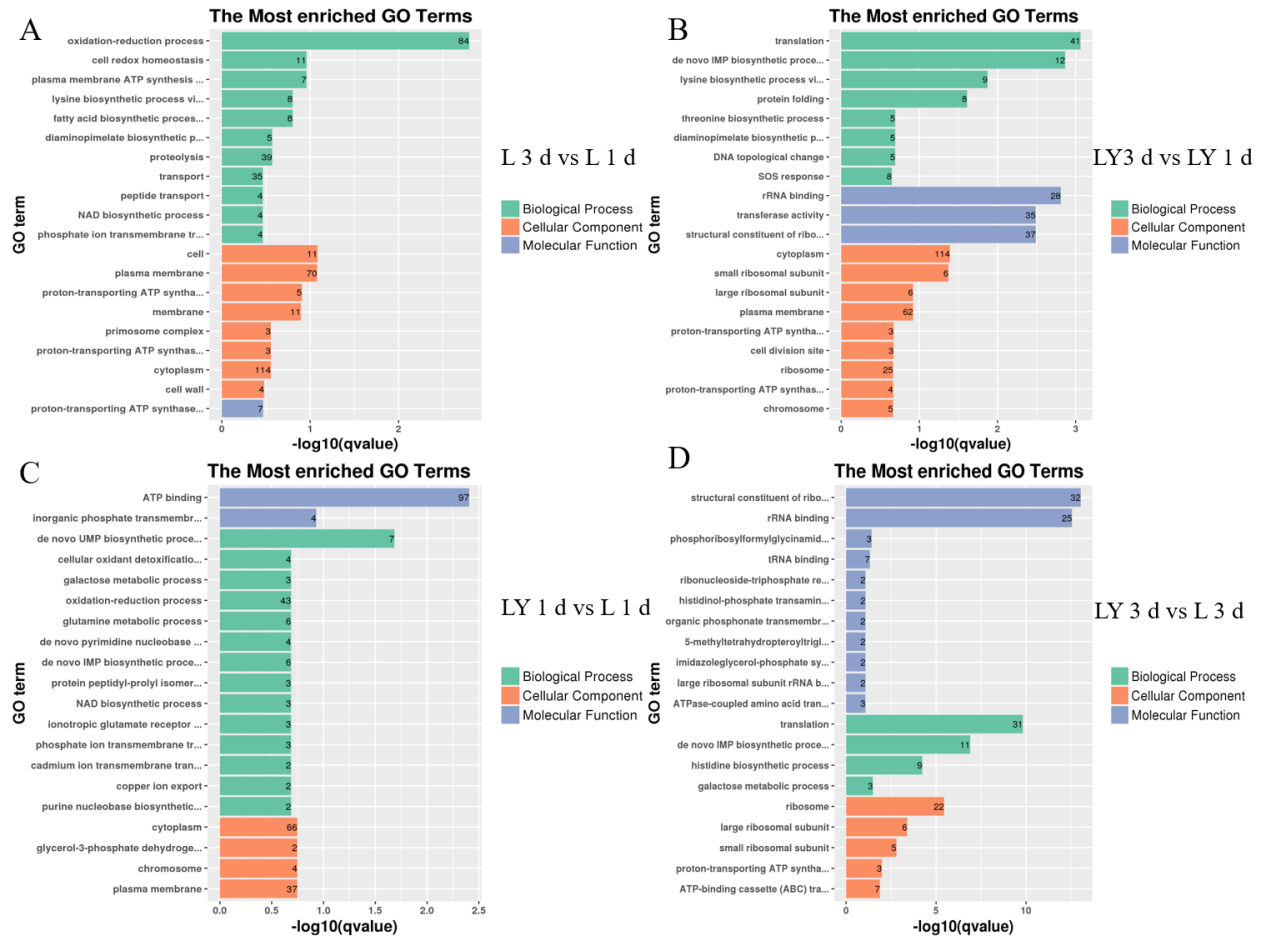


FIGURE S3. GO enrichment analysis of differentially expressed genes in four different experiments (A: L3 d vs L1 d, B: LY3 d vs LY1 d, C: LY1 d vs L1 d, D: LY3 d vs L3 d) inoculated with *L.* *paracasei* (top 20 GO Terms).


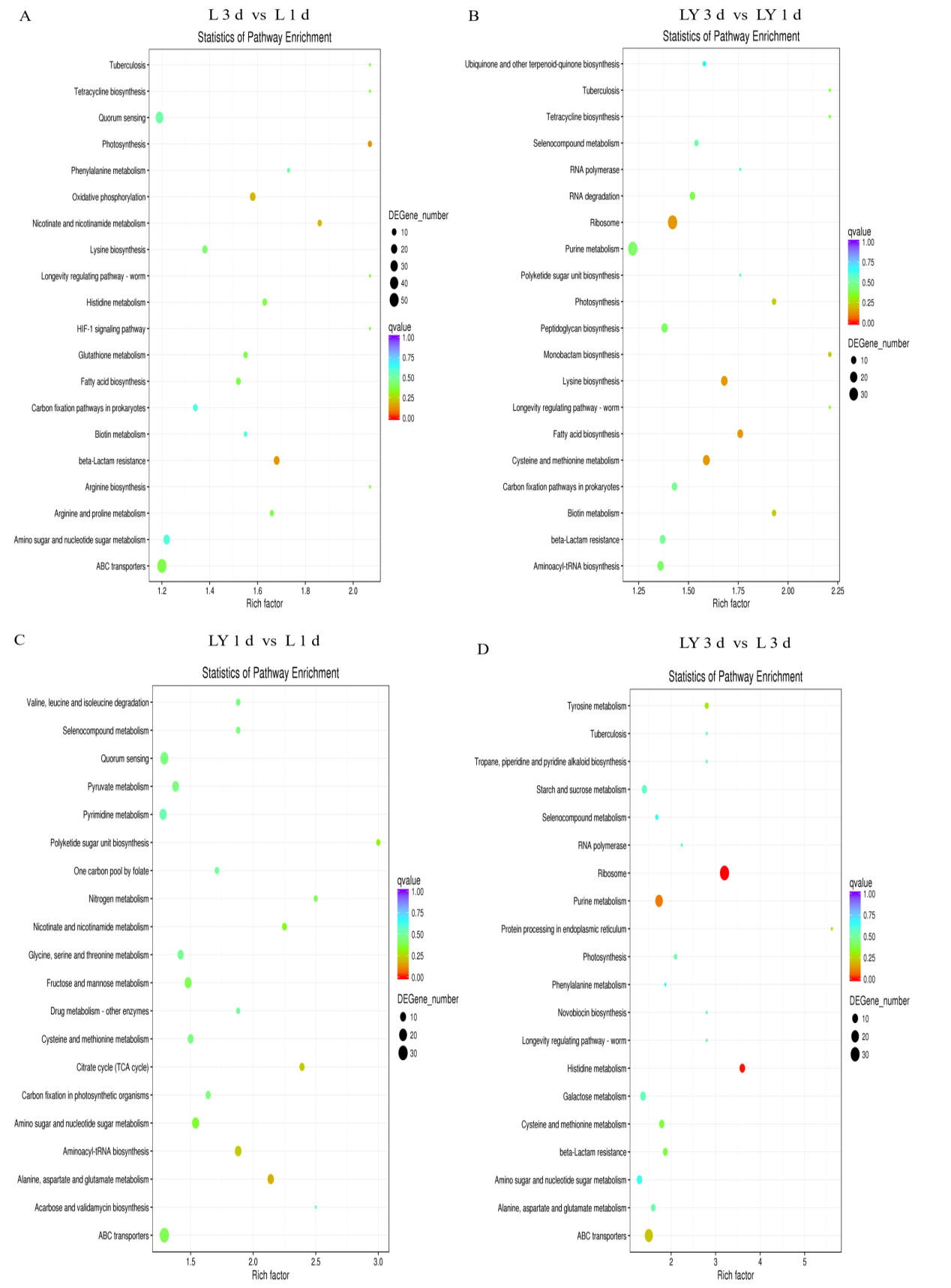


FIGURE S4. Scatter plot of unigenes mapped to the KEGG database. (A) L3 d vs L1 d, (B) LY3 d vs LY1 d, (C) LY1 d vs L1 d, (D) LY3 d vs L3 d. The horizontal coordinates represent the pathway name and the vertical coordinates represents the rich factor. The greater the rich factor, the greater the degree of enrichment. The range of the Q value is [0, 1], and the closer to zero, the more obvious the enrichment. Rich factor, the ratio of the number of genes with significant differences in transcription levels to the total number of all annotated genes in the pathway. Q value, P after correction of the multiple hypothesis test.


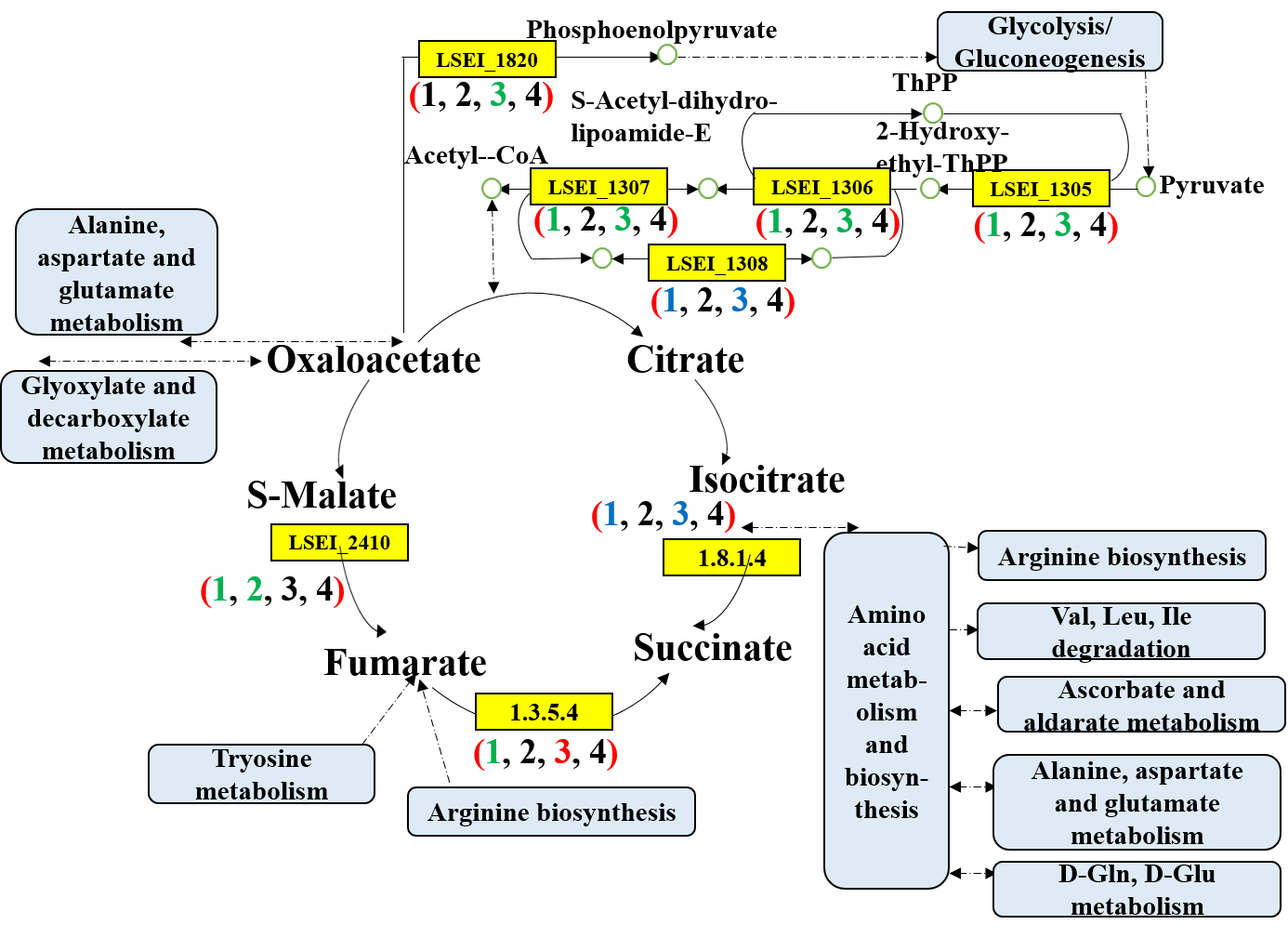


FIGURE S5. Differentially expressed genes of TCA cycle in four pairwise comparisons (Numbers in brackets 1, 2, 3 and 4 refer to the L3 d vs L1 d, LY3 d vs LY1 d, LY1 d vs L1 d and LY3 d vs L3 d, respectively. L1 d refers to single inoculation with *L. paracasei* in first day, L3 d refers to single inoculation with *L. paracasei* in third day, LY1 d refers to mix inoculation with *L. paracasei* and *K. marcianus* in first day, LY3 d refers to mix inoculation with *L. paracasei* and *K. marcianus* in third day. The color of red, green, blue, and black represents the up-regulated gene, down-regulated gene, both of up-regulated gene and down-regulated gene, and no change gene, respectively).
